# Evaluation of a Sexual Transmitted Infection Prevention Program Among University Students in Beira City Central Mozambique: A Study Protocol

**DOI:** 10.3389/frph.2021.745309

**Published:** 2021-10-28

**Authors:** Arlinda Basílio Zango, Rik Crutzen, Nanne de Vries

**Affiliations:** ^1^Faculdade de Ciências de Saúde, Universidade Católica de Moçambique, Beira, Mozambique; ^2^Department of Health Promotion, Faculty of Health, Medicine and Life Sciences, Care and Public Health Research Institute, Maastricht University, Maastricht, Netherlands

**Keywords:** sexual behaviors, evaluation, STI, HIV, prevention, students, Beira, UCM

## Abstract

**Background:** Unhealthy sexual behaviors, such as unprotected sexual intercourse and lack of using screening services increase cyclical transmission of sexually transmitted infections including Human Immunodeficiency Virus (HIV), especially among young adults. Hence health promotion programs can contribute to reduce the consequences, by changing (determinants of) these behaviors. Such interventions need to embrace a comprehensive approach and apply theory-and evidence-based methods. This article describes the protocol for a process and effect evaluation study of a sexually transmitted infection prevention program among university students in Beira city, central Mozambique.

**Methods:** The on-going program at *Universidade Católica de Moçambique* is described following the six steps of Intervention Mapping (IM), with a focus on the evaluation plan (i.e., the final step in IM). The details regarding previous steps in the protocol are briefly described as well, as they lay the foundation for the final step. The overall study will apply a hybrid type 1 approach by assessing the effectiveness of the intervention while gettering implementation. The process evaluation will apply qualitative and quantitative methods to gain insight in the context, reach, dose delivered, dose received and recruitment. Interviews with closed and open-ended questions will be conducted with program implementers and users. A quasi-experimental non-equivalent control group design is used to evaluate the effectiveness. A cohort of university students will be followed for 6 months. Self-administrated questionnaires will be used to collect data every 3 months.

**Discussion:** A combination of process and effect evaluation is proposed. This is a useful and fruitful procedure, since concurrent process evaluation can allow researchers to better interpret findings from the effect evaluation and understand how the intervention might replicate in similar contexts. We decided to follow the IM approach since, it is a theory-and evidence-based, systematic and detailed guide regarding what to do at every steps. A quasi-experimental non-equivalent control group design was chosen to fit the context of the study and generate outcomes with high external validity.

**Study Registration:** 004/CIBS/2020.

## Introduction

Unhealthy sexual behavior leads to sexual transmitted infections (STIs). Global estimation reported about 357 million new cases of the main four curable STIs, namely, chlamydia, gonorrhea, syphilis and Trichomoniase, occurred in people aged 15–49 years in 2012 (1). Also, HIV infections occur but are equally preventable. High prevalence of STIs has been reported in different countries of Africa among young adults (2–4).

A meta-analysis of previous studies revealed an estimated prevalence range for gonorrhea of 6.4–11.6%, chlamydia 3.9–17.8%; trichomoniases 10.5–20.6 and syphilis 2.2–10.3% among youths aged 15–24 years, across countries in the Sub-Saharan region (4). In Mozambique specifically, 82% of women aged 14–20 years were diagnosed with at least one STI (3). National surveillance data revealed an HIV prevalence of 13.2% among the population aged 15–49. In the Sofala province, where Beira city is located, a HIV-prevalence of 16.3% in this age group was found (5).

University students are traditionally young adults within the age range of 17–25 years. This group is faced with trying to adapt to changes in academic workload, support networks, and their new environment, as well as with newly found responsibility due to the greater freedom and control over their lives than ever before. Thus, it is important to recognize that these changes are happening concurrently, affecting their body and mind as well as social relationships, all of which can lead them toward unhealthy or risky lifestyle behaviors, especially regarding sexual behavior (6–8).

Sexual and reproductive health promotion and HIV prevention programs can contribute to preventing the consequences of risky sexual behavior among students. These programs should embrace a comprehensive approach (9). The focus should not only be on abstinence as a behavioral goal, but also include education about the use of condoms, contraceptive methods for sexually active youth as well as seeking health counseling and testing services for STI screening (9, 10).

*Universidade Católica de Moçambique* (UCM) and their partners, assume that young people are facing challenges, such as low self-esteem, lack of control of their own sexuality, unintended pregnancies, acquiring STIs including HIV and sexual abuse (11). Hence, a program for behavior change rooted in the right-based approach to sexuality education is running. The program consists of life skills curricular lessons to first year students, awareness activities addressing sexuality and STI/HIV prevention and provision of counseling and testing service for STI/HIV at all faculties. Unfortunately, the program has not yet been systematically evaluated. In general, scant information and data are available regarding evaluation of such interventions in Mozambique. Therefore, this study will contribute by providing more insight into how such programs targeting university students can be evaluated in a systematic way, using the Intervention Mapping (IM) approach (12).

The overall aim of the present paper is to describe the evaluation study of a sexually transmitted infection prevention program among university students in Beira city, central Mozambique. Specifically, we aim to analyze changes in individual-level and environmental determinants of behavior that in turn influence (un)safe sexual health behavior and the use of STI/HIV screening services among university students in Beira city after exposure to the intervention; and to describe the implementation process. The studies will lead to recommendations for possible improvements in the current and future programs in this region.

## Methods and Analysis

The research is planned to take place in Beira city, Sofala province, which is in the central region of Mozambique. The two study settings are the *Universidade Católica de Moçambique* (UCM) and the *Universidade Licungo* (UniLicungo) Beira extension (functioning as a non-equivalent control group). We have followed the intervention mapping (IM) approach to describe the evaluation of the STI/HIV prevention program among university students that is ongoing at the UCM in the Beira city. IM is an approach to systematically develop, implement and evaluate theory- and evidence-based interventions.

IM consists of six steps: (1) the assessment of the problem; (2) the definition of expected behavioral and environmental outcomes, specifically performance objectives (POs) underlying these outcomes, and matrices of change objectives (CO) resulting from crossing performance objectives with underlying determinants; (3) the definition of the theme, components, sequences, selection of the theory-and evidence-based change methods and the designing of the practical applications delivering these methods; (4) the definition, pretesting and production of messages, materials and protocols; (5) the development of an adoption and implementation plan: finally, (6) the development of an evaluation plan. More details on the IM are available elsewhere (12). Although the focus of the study at hand is on the evaluation plan (i.e., the final step in IM), the details regarding previous steps are briefly described as well, as they lay the foundation for the final step regarding evaluation.

### Evaluation Design

The overall study will apply a hybrid type 1 approach. Hence, the study is to assess effectiveness of the STI/HIV prevention intervention among university students and concurrently collect data on the implementation process to better explain findings and enable the research to answer questions regarding context and fidelity when interpreting the effect results. The process evaluation part concerns a combination of qualitative and quantitative methods. This part is planned to be conducted immediately after setting up the cohort. The main aim is to gain insight in the program implementation process and provide information regarding context, reach, dose delivered and received as well as recruitment to the stakeholders. This information can be helpful in strengthening, improving and disseminating the program to similar settings. The effect evaluation part will apply a quasi-experimental non-equivalent control group design aiming to assess changes in selected (determinants of) behavior(s) and STI/HIV cyclical transmission over time for 6 months.

#### Description of the Program

The program that is being implemented at UCM is described in line with IM to provide insight into the rationale behind the program and how this is used in the evaluation plan.

##### Step 1: Assessment of the Problem

The problem was assessed through epidemiological data available, literature review, review of existing program materials, and six meetings that were held with relevant stakeholders (program implementers and managers of the study settings). Cyclical transmission of STIs including HIV and high prevalence of STI/HIV are public health problems affecting university students, with undesirable consequences such as: increased costs for medical care, reduced life expectancy, infertility and low productivity.

We have selected two behaviors at the individual level (i.e., behavioral factors regarding students) namely: engagement in unprotected sexual practices and unregular seek and use of STI/HIV screening services. These are behaviors that are targeted by the current program and can be changed using theory-and evidence-based methods.

##### Step 2: Program Outcomes and Objectives: Defining Behavioral Outcomes and Performance Objectives - From Logic Model to Matrices of Change Objectives

The expected BOs at the individual level are (a) University students engage in protected sex practices for example consistently and correctly use of condoms; (b) university students regularly seek STI/HIV screening services. Several determinants underlie these behaviors, such as knowledge, risk perception, attitude, self-efficacy, skills and subjective norms. COs specify what needs to be changed (at determinant level) to result in the performance objectives (POs) to be achieved. POs are sub-behaviors resulting in behavioral or environmental outcomes that will lead to better health and improve quality of life of university students. Supplementary File (Appendix 1) presents the matrices of change objectives (including the performance objectives and determinants underlying them) for the program users. To summarize, the expected outcomes are reported correct and consistent use of condom and regular use of STI/HIV counseling and testing services. The study will also measure the personal determinants (knowledge, risk perception, attitude, self-efficacy and subjective norms) of these behaviors.

##### Step 3: Program Design: Intervention Theme, Components, Sequences, Selection of the Theory-and Evidence-Based Methods and Practical Applications

The program consist of three components: (a) trained teachers provide 16 classroom curriculum lessons regarding STI/HIV within the life skills subject for all first year university students at the UCM; (b) volunteer students (activists) engage in sensitizing activities (4 sessions) to increase the use of STI/HIV screening services, and the use of condoms correctly and consistently among university students; and (c) health advisors offer STI/HIV counseling and testing services at all faculties. Methods depicted in Bartholomew Eldredge et al. (12) were used to describe the content of the curricular handbook of students (13, 14), the teachers' manual (13, 15) for the life skills subject, and the national guidelines for STI/HIV screening and treatment (16, 17). The methods were translated into practical applications as described in Supplementary File (Appendix 2), clustered with other intervention components, which were reviewed by two senior investigators in Health Education and Promotion (RC & NdV).

The overarching theme of the program is, Listen, which was translated to the title: “Take the opportunity to listen and to be listened to.” The evaluation will address the three components of the program: (a) trained teachers provide sixteen classroom curriculum lessons regarding STI/HIV within the life skills subject for all first year university students at the UCM; (b) volunteer students engage in sensitizing activities (four sessions) to increase the use of STI/HIV screening services, and the use of condoms correctly and consistently among students, through regular campaign; and (c) health advisors offer STI/HIV counseling and testing services at the faculties.

##### Step 4: Program Development: Identify, Adapt Messages, Materials and Protocols Already Available (see Supplementary File – Appendix 2)

The program has already been developed and activities are planned at the department of Sexual and Reproductive Health, Gender and HIV/AIDS, at the rectory of the UCM (highest administrative level of the university). The implementers' team at Faculty of Health Science consists of one health advisor, three teachers and sixteen volunteer students. At the Faculty of Economy and Management, there is one health advisor, three teachers and sixteen volunteer students. All are committed to deliver the program activities in a collaborative way with the faculty authorities.

###### Teachers' Intervention Activities.

The life skills classes follow a classical method of teaching, through a student-centered learning approach. A teachers' handbook and an equivalent students' handbook are the basic didactic materials. The subject consists of 64 h, of which 34 are devoted to lectures and 30 to practical sessions. Among the 64 h, about 50% are dedicated to SRH contents of which 16 h are committed to address STIs and HIV issues. Yet, the PO and CO defined for the research propose are addressed in sixteen lessons of about 2 h, twice a week, targeting first year graduation students in the first semester at each academic year. The structure and sequence of lessons is guided by the lesson topic translated into specific lesson content (see Supplementary File). For the whole subject, two tests and one exam are performed for the evaluation of student's knowledge. If a student completes the subject with success (marks ≥ 10 out of 20), they are awarded 5 academic credits.

###### Volunteer Students' Intervention Activities.

The volunteer students perform their activities using pamphlets, handbooks, films, posters and flip paper. They also use methods such as interactive lectures, theater followed by a discussion session, and chatting sessions in classrooms. At each session they focus on one issue, for instance, control of their own sexuality and prevention of STIs including HIV. During the evaluation research, volunteer students will provide four sessions of awareness activities following the structure and sequence guided by the lesson topic translated into specific lesson content.

###### Health Advisors' Intervention Activities.

Health advisors offer counseling and testing for STI/HIV services in a private room at each faculty at the UCM; the same will be ensured at the UniLicungo, 3 days per week, from 8:00 to 16:00. The services are delivered according to the national guideline (16), which distinguishes and recommends three main sequential steps of counseling as follows: pre-testing counseling, counseling during the test/examination and post-test counseling.

During a session of counseling, the client discusses with the health advisor issues regarding STIs/HIV acquisition and prevention (for more details see Supplementary File) and can take pamphlets home for continuous consultation and reading. Testing for HIV is performed using Alere DetermineTM HIV-1/2, an *in vitro*, visually read, qualitative immunoassay for the detection of antibodies to HIV1 and HIV 2 in human whole blood of an infected person. If the test detects presence of antibodies, the result is confirmed by Trinity Biotech Uni-GoldTM HIV test, since it is considered the most specific test device; the test is also an *in vitro*, visually read, qualitative immunoassay for the detection of antibodies to HIV.

Additional rapid plasma reagin (RPR) tests (Abbott biokit) will be performed, to screen Syphilis infection for the study purpose. Furthermore, questions will be asked to screen for general symptoms of STIs. The STI diagnosis will be made in accordance with the national guideline. In case of a positive test result or identification of symptoms, the client is referred to the health center for treatment and follow-up if needed.

##### Step 5: Adoption and Implementation Plan

The adoption and implementation plan are presented following the same tasks as in the step 2 to 4; hence, outcomes and objectives (PO and CO) for adoption, implementation and continuation are expressed; methods and practical applications are selected; and an intervention to foster the program adoption, implementation and continuation is developed.

The faculties' managers (program adopters) have decided to adopt the program and the evaluation research by signing the letters of authorization. Thus, the performance objectives for faculties managers are to review the evaluation research materials; provide the program implementers to deliver the lessons, sensitizing activities and screening services; guarantee support from the stakeholders' (decision makers, teachers, volunteer students, health advisor and students); and communicate with the implementers about practice changes in their activities due to the research.

The expected implementation outcomes are (1) Adoption of the program evaluation research “Take the opportunity to listen and to be listened to,” including provision of curricular lessons at UCM, sensitizing activities and screening for STI/HIV at both study settings, and (2) The implementers collaborate in the implementation of the program evaluation research, while are providing intervention activities.

##### Step 6: Evaluation Plan

The framework to guide the evaluation plan was based on program components (12, 18–21). Hence, process evaluation and effect evaluation will be conducted concurrently as described in the following sections.

#### Process Evaluation

The process evaluation including its indicators are summarized in Table 1. It provides information to understand and inform the stakeholders about context, reach, dose, fidelity and recruitment during activity performance (22) by understanding the program process implementation. Specifically, describing the program process delivery in terms of context, reach, dose delivered and received, fidelity and recruitment; assess implementers' opinion regarding improvement of the program and describe implementers' opinions regarding pros and cons of the program.

**Table 1:** Process evaluation overview for the “Take the opportunity to listen and to be listened to!”

**Domain of evaluation**	**Intervention component**	**Questions/purpose**	**Indicators**	**Methods**
**Context**	Life skills lessons	Has the context changed for STI/HIV prevention program during the intervention implementation? What changes have been made in curricular lessons provision?	Classroom conditions for group discussion activities, design/schemes for group activities. Students' criterion to attend life skills lessons. Training certificate.	Observation of classroom and handbooks. UCM guideline and police tracking. Interview to teachers.
	Awareness for STI/HIV prevention	What changes have been made in awareness activities delivery?	Manual of sensitizing activities. Training certificates.	Observation and Interview to a sample of activists.
	Counseling and testing services	What changes have been made in STI/HIV screening offering services?	Guideline for STI/HIV screening and Standard Operation Procedures. Training certificate.	Observation. Interview to heath advisor.
**Reach**	Life skills lessons	To what extent curricular lessons are reaching anticipated participants?	Percentage of first year students who are targeted by life skills lessons in the first semester on the academic year.	Determination of participation rates of students in the life skills classes. Interview to teachers of life skill.
	Awareness for STI/HIV prevention	To what extent awareness activities are reaching anticipated participants?	Percentage of first year students who are targeted by awareness activities in the first semester on the academic year.	Determination of participation rates of students in the awareness activities. Interview to activist.
	Counseling and testing services	To what extent counseling and testing for STI/HIV services reach anticipated participants? Are unanticipated groups taking part? Whom are these groups? Are any groups being missed?	Percentage of university students who use STI/HIV screening services. Percentage of users who are not university students.	Determination of monthly visit rates of students to the health advisor. Interview to health advisor.
**Dose delivered**	Life skills lessons	How much of the life skills contents related to STI/HIV prevention are being delivered? What is being omitted or delivered inconsistently? Why?	Percentage of lessons provided, and average of time length of each scheduled lesson.	Book-lessons record, and checklist or time record for lessons deleverage.
	Awareness for STI/HIV prevention	How much of the awareness activities are being delivered? What is being omitted or delivered inconsistently? Why?	Percentage of sensitizing sessions delivered, and average of time length for each session.	Records of sensitizing activities and time record sheet.
	Counseling and testing services	How much of the counseling and testing contents are being delivered? What is being omitted or delivered inconsistently? Why?	Percentage of screening services offered per month.	Checklist.
**Dose received/ participants responsiveness**	Life skills lessons	What is the average dose received by lesson (attendance per lesson)? What parts of the life skills addressing STI/HIV prevention are not received consistently? Why? How do the participants appreciate contents of life skills addressing STI/HIV prevention?	Percentage of 1st year students attending lessons at each scheduled time. Participation/interest Narrative reports from interviewers.	Attendance record. Interview to teachers. Interview to intervention users. Observation, interview to teachers, focus group interview to intervention users.
	Awareness for STI/HIV prevention	What is the average dose received by awareness activity? What parts of the awareness activity are not received consistently? Why? How do the participants appreciate contents of awareness activities regarding prevention of STI/HIV?	Percentage of intended participants attending each session of sensitizing activity. Participation/interest. Narrative reports from interviewers.	Analysis of attendance record for each session of sensitizing activities. Observation, interview to activists, focus group interview to intervention users.
	Counseling and testing services	What is the average dose received by intervention (participants for each component)? What parts of the intervention components are not received consistently? Why? How do the participants appreciate counseling and testing sessions?	Percentage of university students who visit STI/HIV screening service per month over the evaluation period. Narrative reports from interviewers.	Review and analysis of counseling records. Interview to selected sample of users Interview to health advisor and focus group interview to intervention users.
**Fidelity**	Life skills lessons	Have the theory and evidence-based change methods been appropriately implemented in the delivering of life skills lessons?	Degree to which life skills content of each lesson are linked to the theoretical methods and practical application to specific determinants.	Analysis of the lesson plans, observation and checklist. Interview to a sample of intervention user.
	Awareness for STI/HIV prevention	Have the theory and evidence-based change methods been appropriately implemented during awareness sessions?	Degree to which awareness content of each session are linked to the theoretical methods and practical application to specific determinants.	Narrative description of observed activities, and Checklist. Interview to a sample of intervention user.
	Counseling and testing services	Have the theory and evidence-based change methods been appropriately implemented during counseling and testing provision?	Degree to which counseling and testing content of each session are linked to the theoretical methods and practical application to specific determinants.	Interview to a sample of intervention user.
**Recruitment**	Life skills lessons	How are participants attracted to life skills lessons?	Timetable.	Tracking of timetable.
	Awareness for STI/HIV prevention	How are participants attracted to awareness activities?	Plans of awareness activities.	Tracking of awareness plans.
	Counseling and testing services	How are participants attracted to visit health advisor?	Awareness material.	Observation of where and what material are placed in public places.

Qualitative and quantitative approaches will be combined for the process evaluation, combining documents and records review, as well as interviews with the program implementers and users, shortly after setting up the effect evaluation cohort, and during the study period. Additionally, focus group interviews are planned, aiming to explore experiences, barriers that students encounter to perform recommended sexual health behaviors and to contextualize the findings of the effect evaluation part.

##### Sample Size and Sampling for the Process Evaluation

All teachers involved in teaching life skills are included, health advisors and a sample of activists will be invited to take part in the study. Additionally, A sample of first year students from the Faculty of Health Sciences and the Faculty of Economy and Management, both at the UCM, will be systematically selected through a stratified random sampling procedure strategy from the cohort's participants list within faculty and gender groups, as well as consideration of a number of interviews to achieve saturation, which is expected to range between 6 and 12 per gender and per faculty (23–25). The same procedure will be applied to select participant from the activists' groups at both Faculties of the UCM.

##### Study Procedure

Inclusion criteria:

**Activists**

- Attending second to fourth year for all courses, and second to sixth year for medicine at the UCM.
- Actively engaged in sensitizing activities.

**Health advisors**

- Holding a valid fulltime working contract at the UCM.
- Formally assigned to offer STIs screening, counseling and testing services at the UCM.

**Teachers**

- Holding a valid fulltime working contract at the UCM.
- Assigned to teach the life skills discipline in the current academic year.

**Program users**

- First year university students from UCM.
- Being aged 16 to 25 years old.

###### Technic and Instruments of Data Collection.

The process evaluation research will use different techniques and instruments of data collection (see Table 1). Summarizing, face-to-face structured interviews will be applied to collect data from program implementers. The interviews will follow a structured interview guide (Appendix 4a–c). The instruments will be pre-tested for language adjustment, and revision will be performed if needed.

Observations and checklists will also be used to assess provision of curricular lessons and delivery of awareness activities. Tracking of program materials, training records, lessons, awareness activities and screening records will be used to capture data regarding reach, dose delivered and dose received, including fidelity and recruitment. Moreover, face-to-face structured interviews (Appendix 4d) and focus group interviews (Appendix 4e) will be conducted with a sub-sample of users to assess participants' responsiveness to the intervention.”

###### Collection of Data.

The first interview will be done to assess how activists engage in sensitization activities, what they think about the usefulness of the program for themselves and for other students, and what they think about improvement of the intervention. The interview will be conducted face-to-face using a structured guide (Appendix 4a).

The second interview is designed for teachers of the life skills subject, to learn more about the area of their training, how they were selected to teach the subject, how long they have been working as university lecturers, what their experience with SRH contents is (including number of trainings relative to the field), how they evaluate the attendance and participation of students to lessons of this subject. The interview will be guided by a structured form (Appendix 4b).

The last interview among the implementers will be done among the health advisors; open-ended questions (Appendix 4c) will be used to seek data regarding their area of training, experience and skills as health advisor (including trainings on SRH), what they think about utility of their activities to students, what sort of problems students usually report when they visit them, what the main challenges in daily work are, and what they propose to improve the program.

All implementers will be asked to fill in a checklist and record sheets, in order to evaluate the reach, dose delivered and received, recruitment and fidelity of the program implementation. During the interview process, the conversations will be recorded and subsequently transcribed to limit possible bias and ensure natural information provided by the participant; also the interviewers will take notes during the interaction.

Additionally, a selected sample of program users will be asked to attend a face-to-face interview. The interview will be administered using a structured interview guide (Appendix 4d). At the end of the follow-up period, focus group interviews are planned, aiming to explore experiences, motivations and barrier of students to engage in recommended sexual health behaviors. The interview will be administered by study data collectors using a structured interview guide (Appendix 4e).

Focus group interviews will enable to access person's experiences, beliefs, opinions, values and desires. The sampling procedure will follow the same design as described above. Hence, from the list of participants studying at the Faculty of Health Sciences and Faculty of Economy and Management at the UCM, subjects will be randomly selected within gender groups and asked to take part of the group interview, until the sample size is completed (four groups of 6–12 participants) and saturation is achieved.

Participants will receive written and oral information about the group interview details (Appendix 4e), including assurance regarding ethical issues (anonymity and confidentiality); the participant will be given enough time to decide. If he/she decides to take part in the focus group interview, the data collector will schedule the date and time for interview according to the participant preference.

Written informed consent process will be conducted with all participants before the interview. Confidentiality and anonymity will be ensured to the participant, thus, the interview contents will be labeled using codes, and it will take place in an area free from distractions and at the times and location that are considered suitable by the participants, for instance, at the faculty in a closed classroom. The focus group interview may last 1.5–2 h and will be carried out within gender and faculty specific groups. Hence, four focus group interviews are expected to be held: each gender group within each of the two faculties.

All interview sessions will be recorded and transcribed afterward. Two researchers will be present, one as a moderator and another one as observer. The observer will not be allowed to contribute with any idea, his/her task will be to take notes and record the sessions in as much detail as possible. The participants will be explained the procedure before the interview starts. All focus group interview participants will be compensated for transport, and snacks will be shared at the end of the interview.

##### Data Analysis

Quantitative data will be processed and analyzed using SPSS version 25 (Statistical Package for the Social Sciences); descriptive statistics and proportions will be reported.” Qualitative data will be managed and analyzed using the NVivo software. A combination of thematic method and narrative analysis (26–31) will be performed. On the one hand, the thematic approach will consist of transcription of the recorded interviews, coding, identification of categories and building theme maps where each category fits and interpret the findings based on themes that represent experiences, beliefs, attitude and other constructs regarding the behavior.

On the other hand, the narrative approach will consist of understanding the content and structure of the transcribed stories through reading and re-reading the transcriptions, identify crucial constructs (feeling, reactions, accounts, excuses, explanations), writing a summary addressing the main elements such as beginning, middle and the end of the narrative. The transition between themes will be captured and thematic connection of ideas will be developed based on the content of the story, finally comparison among different views of participants regarding the intervention will be presented.

#### Effect Evaluation

The effect evaluation including its indicators and methods are summarized in Table 2. The main questions to be answered are: (a) What changes in behavior and individual-level determinants occurred, and (b) To what extent is the students use correctly and consistently condom, use screening services regularly as well as the prevalence and incidence of STI/HIV changed? The hypotheses are: (a) The program is effective in positively changing personal determinants (Knowledge, risk perception, attitude, self-efficacy, skills and subjective norms) associated with unprotected sexual intercourse behavior among university students and regular use of screening services for STI/HIV; (b) The program is effective in reducing cyclical transmission of STI/HIV among university students; (c) The program is effective in increasing the use of STI screening services; (d) The program is effective in increasing reported rate of condom use among participants.

**Table 2:** Effect evaluation overview for the “Take the opportunity to listen and to be listened to!”

**Outcome**	**Question/Prepose**	**Indicator**	**Methods**
Individual-level determinants of safe sex	What changes in individual-level determinants of safe sex occurred?	Likert-type response scales means and standard deviation.	Questionnaire to a sample of first year university students from intervention and control group.
Individual-level determinants of STI/HIV counseling and testing services use	What changes in individual-level determinants of STI/HIV counseling and testing services use occurred?	Likert-type response scales means and standard deviation.	Questionnaire to a sample of first year university students from intervention and control group.
Reported correct and consistent use of condom	To what extent the students use correctly and consistently condom?	Proportion of students who express correct use of condom and/or report use of condom.	Questionnaire to a sample of first year university students from intervention and control group.
Use of STI/HIV counseling and testing services	To what extent the students use STI/HIV screening services regularly?	Proportion of students who express regular use of screening services. Proportion of students who regularly use Screening services.	Questionnaire to a sample of first year university students from intervention and control group. Analysis of counseling and testing services records.
Occurrence of STI/HIV	To what extent the prevalence and incidence of Syphilis, HIV and reported general symptoms of STI changed.	Prevalence and incidence of Syphilis/HIV or symptoms of any STI.	Analysis of counseling and testing services records, including test results.

A cohort study applying a quasi-experimental non-equivalent control group design with pre-post approach will be used since the intervention is ongoing and by its nature, the use of a full experimental design is unjustifiable. Students registered for the first year, willing to take part in this study, will fill in the questionnaire at enrollment, post intervention and at follow up. The participants will be encouraged to visit a health advisor to undergo an STIs screening through clinical and testing examination. Considering that the prevalence of STI in general is high among young adults, and the primary outcome of the intervention is (determinants of) behavior change, all students, no matter their STI/HIV state, will be enrolled in the cohort and followed-up for 6 months. Students enrolled at UCM will be exposed to the full program, and students from UniLicungo will only receive information from research team and teachers to visit STI counseling and testing office.

The main aims of the cohort are: To analyze changes in behavior and individual-level determinants that in turn influence unprotected sex behavior and use of STI/HIV screening services among university students in Beira city after exposure to the intervention, as well as to monitor cyclical transmission of STIs including HIV among first year university students in Beira at 3 and 6 months after setting up the cohort. During the follow-up period there will be regular consultations, every 3 months for STIs/HIV screening and questionnaire self-filling. Treatment will be offered at the health center to students that are diagnosed with an STI, and the participant will not be withdrawn from the cohort.

##### Sample Size and Sampling

At baseline, clustered and random sampling will be applied. University students will be enrolled consecutively from randomly selected classes (clusters) at the UCM and UniLicungo. Considering that the baseline survey will serve as identification and enrolment point of participants to the cohort, and the main purpose is to look for differences on determinants and behavior changes between groups (students exposed to the full program and those not exposed); based on a 95% confidence interval, desired confidence interval half-width of 0.15 and Cohen's d of 0.2, a total of 687 participants are needed (32). Assuming a 20% dropout, the final sample size is 824. Hence, the goal is to include 412 participants per university. At each university, ten classes (graduation programs) will be randomly selected, and study participants will be enrolled from selected classes until the sample size is completed starting from the first class randomly identified.

Inclusion criteria

- First year university students studying at one of the two universities in faculties located in Beira city.
- Being aged 16–25 years old.
- Having residency in Beira city for the entire academic year.

##### Study Procedure

Data collection will be performed by three data collectors after being trained in a 2-day session. The training will focus on study aims, how to obtain informed consent, how to approach the participants and how to distribute and collect the questionnaires. The data will be collected through a self-administered structured questionnaire (see Supplementary File, Appendix 3) with closed-ended questions in Portuguese, the official language of Mozambique. The questionnaire is developed based on the change objectives (see matrices in Supplementary File), using Likert-type response scales (33) and guided by recommendations of Fishbein & Ajzen (34). One or two days before data collection, courses' coordinator of each selected class will explain about the study and invite students to take part. Confidentiality will be ensured, and codes will be used to identify each participant's questionnaire including the study participant's identification card.

Informed consent will be obtained collectively before questionnaire distribution to the class though each candidate will freely decide and sign the consent form. The data collection process will take place in classrooms. The same questionnaire will be administrated to the study participants three times (time 0 at enrolment visit; time one after 3 months of program implementation and time 2 6 months after). At the time one and two, participants will be asked to respond only to questions regarding intersexual relationship (section 2) and questions addressing behavioral outcome 1 and 2 (section 3 and 4, respectively).

During the time of filling in questionnaires, students will not be allowed to chat or to look at classmates' answers. Thus, in case of doubt, students will be advised to ask the data collector for clarification. After a given time for completing, the data collector will recollect the questionnaires, give a study participant identification card, and recommend her/him to visit the health advisor office for STIs counseling and screening within the following days.

The health advisor will offer services according to the national guideline (17). He/she will also record the results of STI/HIV screening in the specific form with participant identification number (code). A standard operation procedure (SOP) will be provided to the health advisors. The health advisors will fill in a checklist for each client assisted, they will also be asked to fill in the client record sheet with required data from all clients regardless of being a study participant.

All quantitative data will be processed and analyzed using SPSS (Statistical package for Social Sciences, V. 25), descriptive statistics and proportions will be reported. The baseline data will be compared using t- tests and Chi-square tests. After intervention, data from different universities will be compared using multilevel regression models considering the baseline values and nesting of students within classes. Multilevel regression model analysis has been referred to be an eligible method to analyze data from research with a hierarchical structure, and variables measured at different levels and time-points (35–38). This method enables an assessment of the effects of variables measured at different levels of the hierarchy, for example class, student and repeated points of measure indeed, many benefits has been mentioned (36). The data from the baseline survey will be used to estimate the effect size. Additionally, Cohen's d test will be used to estimate the effect size with 95% confidence interval, relative risk and Odds ratio will be determined to measure the extent to which the intervention affects the (determinants of) behavior.

## Discussion

Evaluation of health promotion programs is seen as an important step in intervention development, because it enables to learn about the effects of interventions and how they are established (22), indeed it opens up the “black box” of the intervention (39). In this study, a combination of process and effect evaluation is proposed. This is a useful and fruitful procedure, since concurrent process evaluation can allow researchers to better interpret findings and understand how the intervention might replicate in similar contexts. Nevertheless, the method can limit biases in estimating effects, while building a detailed comprehension of causality (22) through correlation of process indicators with outcomes (40) to support stakeholders and scientific audience in interpreting the reports.

The result of this study will inform and guide the development of optimal sexual and reproductive health behavior promotion programs targeting university students, using a systematic approach. Having assessed the natural conditions under which intervention targeting STI/HIV prevention issues among university students works, the findings will support public health decision makers to expand the program to other universities (if deemed appropriate) and make adaptions (where needed).

There are many approaches that can be applied to develop, implement and evaluate health promotion interventions, from the narrow one to the most comprehensive such as the Behavior Change Wheel and Intervention Mapping. O'Cathain end colleagues describe eight categories: 1. Partnership approaches; 2. Target population-centered; 3. Theory and evidence-based, 4. Implementation-based, 5. Efficacy based; 6. Stepped based; 7. Intervention-specific and 8. Combination (41). Our study belongs to category 3.

We decided to follow the IM since it is a theory-and evidence-based, systematic and detailed guide regarding what to do, especially on how to perform specific needed activities to achieve intervention transparency (39, 41, 42). Although the approach is time and resource consuming, it is a helpful method which enables the researchers to receive systematized support, while planning and concretizing aims including the whole intervention, from all stakeholders (43, 44).

The program described in this manuscript, which we aim to evaluate, is already in its full implementation, meaning that it might be an unjustifiable and unfeasible procedure to apply a full experimental study design with random allocation to intervention and control group, which would imply excluding some classes or students from the intervention at UCM, while they share the same environment and policies. Hence to have a comparison group, similar in age and level of education, a quasi-experimental design assigning students from another university as comparison group is a rational and acceptable approach to evaluate the effect of the intervention aiming to change (determinants of) behavior with limited biases.

Traditionally, randomized controlled trials have been seen as gold standard for generating casual evidence in health sciences field, especially when it comes to warrant inclusion criteria in systematic reviews and meta-analysis (45, 46). However, for this study, a quasi-experimental non-equivalent control group design was chosen, since the design combines some of the advantages of the full experimental designs with those of no experimental studies, offering distinct advantages compared to randomized controlled trials. Particularly, the design limits some of the threats to external validity that can be seen as a weakness of the full experimental designs (45).

Although quasi-experimental studies have some advantages compared to other designs, it is recognized that they will only result in acceptable or valid inferences when conducted in a way that meet certain assumptions (45). They are recommended when the study pretends to evaluate complex interventions for instance health behavior change (46–48), which is our case. The design will enable to generate outcomes with high external validity since the data will be collected under natural conditions. Admittedly, quasi-experimental study designs as described above, are challenged by some limitations such as lack of individual participant randomization to each arm and need for advanced statistical analysis. The results from this study can be influenced by several pitfalls for instance, selection bias, maturation bias, Hawthorne effect (observation effect), historical bias, regression to mean, ascertainment bias and reporting bias (49–51). Most of these biases will be controlled by using the control group pre-post approach.

## Ethics Statement

The studies involving human participants were reviewed and approved by Interinstitutional Committee on Bioethics of Health - CIBS Sofala. IRB00002657. Written informed consent to participate in this study was provided by the participants' legal guardian/next of kin.

## Author Contributions

AZ and RC developed and conceived the research protocol manuscript. NV contributed to the study design and reviewed the first draft of the paper. All authors contributed to the article and approved the submitted version.

## Funding

AZ is supported by the Nuffic, NICHE Program grant number: 30606015N, led by the Maastricht University as scholarship for her PhD trajectory. Obtaining this scholarship did not include peer-review of the study protocol.

## Conflict of Interest

The authors declare that the research was conducted in the absence of any commercial or financial relationships that could be construed as a potential conflict of interest.

## Publisher's Note

All claims expressed in this article are solely those of the authors and do not necessarily represent those of their affiliated organizations, or those of the publisher, the editors and the reviewers. Any product that may be evaluated in this article, or claim that may be made by its manufacturer, is not guaranteed or endorsed by the publisher.

## Abbreviations

AIDS: Acquired Immunodeficiency Virus
BO: Behavior outcome
CDC: Center for Disease Control
CIBS: Comité Interstitucional de Bioética de Sofala
CO: Change Objective
DNAM: Direcção Nacional de Assistência Médica
HIV: Human Immunodeficiency Virus
IM: Intervention Mapping
MISAU: Ministério da Saúde
NdV: Nane de Vries
PO: Performance objectives
RK: Rik Krutzen
RPR: Rapid Plasma Reagin
SOP: Standard Operation Procedures
STI: Sexually Transmited Infection
UCM: Universidade Católica de Moçambique
UM: Maastricht University
UniLlicungo: Unversidade Licungo.

## References

[1] Newman L Rowley J Hoorn S Vander Wijesooriya NS. Global Estimates of the Prevalence and Incidence of Four Curable Sexually Transmitted Infections in 2012 Based on Systematic Review and Global Reporting. PLoS ONE. (2015) 10:e0143304. 10.1371/journal.pone.0143304 26646541 PMC4672879

[2] Francis SC Mthiyane TN Baisley K Mchunu SL Ferguson JB Smit T . Prevalence of sexually transmitted infections among young people in South Africa: A nested survey in a health and demographic surveillance site. PLoS Med. (2018) 15:1–25. 10.1371/journal.pmed.1002512 29485985 PMC5828358

[3] Menéndez C Castellsagu X Renom M Sacarlal J Lloveras B Klaustermeier J . Prevalence and risk factors of sexually transmitted infections and cervical neoplasia in women from a rural area of southern mozambique. Infec Dis Obstet Gynecol. (2010) 2010:1–9. 10.1155/2010/609315 20706691 PMC2913799

[4] Torrone EA Morrison CS Chen P Kwok C Francis C Hayes RJ . Prevalence of sexually transmitted infections and bacterial vaginosis among women in sub- Saharan Africa : an individual participant data meta-analysis of 18 HIV prevention studies. PLoS Med. (2018) 15:1–38. 10.1371/journal.pmed.1002511 29485986 PMC5828349

[5] INE; MISAU. Inquérito de Indicadores de Imunização, Malária e HIV/SIDA. Maputo: INS (2018).

[6] Ah DV Ebert S Ngamvitroj A Park N Kang D-H. Predictors of health behaviours in college students. J Adv Nurs. (2004) 48:12. 10.1111/j.1365-2648.2004.03229.x 15533084

[7] Cardoso BAP Santos M leonardo SC Berardinelli LMM. A relação estilo de vida e tabagismo entre acadêmicos de enfermagem. Rev Electrónica Enfermagem. (2009) 11:368–74. 10.5216/ree.v11.47019

[8] Wang D Xing X Wu X. Healthy lifestyles of university students in China and influential factors. Sci World J. (2013) 2013:1–10. 10.1155/2013/412950 23935418 PMC3727084

[9] Santelli JS Kantor LM Grilo SA Speizer IS Lindberg LD Heitel J. Abstinence-only-until-marriage: an updated review of U.S. Policies and programs and their impact. J Adol Health. (2017) 61:273–80. 10.1016/j.jadohealth.2017.05.031 28842065

[10] Black M Basile K Breiding M Smith S Walters M Merrick M . The National Intimat Partner and Sexual Violance Survey (NISVS): 2010 Summary Report. Atlanta, GA: National Center for Injury Prevention and Control, Centers for Disease Control and Prevention (2011).

[11] UCM UM. Capacity Building for Innovative Interventions and Services on Sexual and Reproductive Health and Rights. Beira: Univesidade Católica de Moçambique.

[12] Bartholomew Eldredge K Markham C Ruiter R Fernández M Kok G Parcel G. Planning health promotion programs. In: Jossey-Brass, editor. An Intervention Mapping Approach. 4th ed. San Francisco, CA: Jossey Bass (2016).

[13] Berg Y van den Sitoe L Laissone EC Impissa N da C Matinada R Chuva L . Habilidades de Vida, Saúde Sexual e Reprodutiva, Género e HIV. Manual de Estudante. Beira: Universidade Católica de Mocambique (2020). p. 1–48.

[14] Tengler H Laice E. Habilidades de Vida, Saúde Sexual e Reproductiva, Género e HIV&SIDA. Manual do Estudante. Beira: Universidade Católica de Mocambique (2015).

[15] UCM. Habilidades de Vida, Saúde Sexual e Reproductiva, Género e HIV&SIDA. Manual do Docente. Beira: Univesidade Católica de Moçambique (2015). p. 1–51.

[16] MISAU/DNAM. Directriz Nacional Para a Implementação do Aconselhamento e Testagem em Saúde. Maputo: Ministério da Saúde (2015).

[17] MISAU. Tratamento Antirretroviral e Infecções Oportunistas do Adulto, Adolescente, Grávida e Criança. Maputo: Ministério da Saúde (2016). p. 24–5.

[18] CDC. Sexually Transmitted Disease Surveillance 2016. Atlanta, GA: U.S. Department of Health and Human Services (2017).

[19] Grossmeier J Terry PE Cipriotti A Burtaine JE. Best practices in evaluating worksite health promotion programs. Am J Health Promot. (2010) 24:1–12. 10.4278/ajhp.24.3.TAHP 20073390

[20] Saunders RP Evans MH Joshi P. Developing a process-evaluation plan for assessing health promotion program implementation: a how-to guide. Health Promot Pract. (2005) 6:134–47. 10.1177/1524839904273387 15855283

[21] Wimbush E Watson J. An evaluation framework for health promotion: theory, quality and effectiveness. Evaluation. (2000) 6:301–21. 10.1177/135638900000600302

[22] Moore GF Audrey S Barker M Bond L Bonell C Hardeman W . Process evaluation of complex interventions: Medical Research Council guidance. BMJ. (2015) 350:h1258. 10.1136/bmj.h1258 25791983 PMC4366184

[23] Guest G Bunce A Johnson L. How many interviews are enough? An experiment with data saturation and variability. Field Meth. (2006) 18:59–82. 10.1177/1525822X05279903

[24] Palinkas LA Horwitz SM Green CA Wisdom JP Duan N Hoagwood K. Purposeful sampling for qualitative data collection and analysis in mixed method implementation research. Administrative policy. Mental Health Serv Res. (2015) 42:533–44. 10.1007/s10488-013-0528-y 24193818 PMC4012002

[25] Robinson OC. Sampling in interview-based qualitative research: a theoretical and practical guide. Qualit Res Psychol. (2014) 11:25–41. 10.1080/14780887.2013.801543

[26] Bowling A Ebrahim R. Handbook of Health Research Methods Investigation, Measurement and Analysis. Open University Press (2005).

[27] Burnard P Gill P Stewart K Treasure E Chadwick B. Analysing and presenting qualitative data. Br Dent J. (2008) 204:429–32. 10.1038/sj.bdj.2008.292 18438371

[28] Castleberry A Nolen A. Thematic analysis of qualitative research data: is it as easy as it sounds? Curr Pharm Teach Learn. (2018) 10:807–15. 10.1016/j.cptl.2018.03.019 30025784

[29] Earthy S Cronin A. Narrative analysis. In: Gilbert N, editor. Research Social Life. 3rd ed. London: Sage (2008).

[30] Gray DE. Doing Research in the Real World. 4th ed. In: Seaman J Fila C Statham C Bedford T, editors. London: SAGE (2018).

[31] Liamputtong P. Qualitative data analysis: conceptual and practical considerations. Heal Promot J Aust. (2009) 20:133–9. 10.1071/HE09133 19642962

[32] Peters G-JY Crutzen R. Knowing exactly how effective an intervention, treatment, or manipulation is and ensuring that a study replicates: accuracy in parameter estimation as a partial solution to the replication crisis. Psychol Health. (2020) 0:1–19. 10.1080/08870446.2020.1757098 32378428

[33] Vagias W. Likert-Type Scale Response Anchors. Clemson University; Clemson International Institute for Tourism & Research Development, Department of Parks, Recreation and Tourism Management (2006). p. 3–4.

[34] Fishbein M Ajzen I. Predicting and Changing Behavior: The Reasoned Action Approach. New York, NY: Library of congress catologing-in-publication data (2010).

[35] Austin PC Goel V Van Walraven C. An introduction to multilevel regression models. Can J Public Heal. (2001) 92:150–4. 10.1007/BF03404950 PMC6979737 11338155

[36] Leppink J. Data analysis in medical education research: a multilevel perspective. Perspect Med Educ. (2015) 4:14–24. 10.1007/s40037-015-0160-5 25609172 PMC4348225

[37] Leppink J. Analysis of covariance (ANCOVA) vs. Moderated regression (MODREG): why the interaction matters. Heal Prof Educ. (2018) 4:225–32. 10.1016/j.hpe.2018.04.001

[38] Twisk J Bosman L Hoekstra T Rijnhart J Welten M Heymans M. Different ways to estimate treatment effects in randomised controlled trials. Contemp Clin Trials Commun. (2018) 10:80–5. 10.1016/j.conctc.2018.03.008 29696162 PMC5898524

[39] Kok G Mesters I. Getting inside the black box of health promotion programs using intervention mapping. Chronic Illn. (2011) 7:176–80. 10.1177/1742395311403013 21900338

[40] Baranowski T Stables G. Process evaluations of the 5-a-day projects. Baranowski and stables. Heal Eval Behav. (2000) 27:157–66. 10.1177/109019810002700202 10768797

[41] O'Cathain A Croot L Sworn K Duncan E Rousseau N Turner K . Taxonomy of approaches to developing interventions to improve health: a systematic methods overview. Pilot Feasibility Stud. (2019) 5:1–27. 10.1186/s40814-019-0425-6 30923626 PMC6419435

[42] Koutoukidis DA Lopes S Atkins L Croker H Knobf MT Lanceley A . Use of intervention mapping to adapt a health behavior change intervention for endometrial cancer survivors: the shape-up following cancer treatment program. BMC Public Health. (2018) 18:1–10. 10.1186/s12889-018-5329-5 29587699 PMC5869761

[43] Dumas A-A Lemieux S Lapointe A Provencher V Robitaille J Desroches S. Development of an evidence-informed blog to promote healthy eating among mothers: use of the intervention mapping protocol. JMIR Res Protoc. (2017) 6:e92. 10.2196/resprot.7147 28526669 PMC5457529

[44] Kobel S Wartha O Wirt T Dreyhaupt J Lämmle C Friedemann EM . Design, implementation, and study protocol of a kindergarten-based health promotion intervention. Biomed Res Int. (2017) 2017:1–9. 10.1155/2017/4347675 28303253 PMC5338306

[45] Bärnighausen T Tugwell P Røttingen JA Shemilt I Rockers P Geldsetzer P . Quasi-experimental study designs series—paper 4: uses and value. J Clin Epidemiol. (2017) 89:21–9. 10.1016/j.jclinepi.2017.03.012 28365303

[46] Tarquinio C Kivits J Minary L Coste J Alla F. Evaluating complex interventions: perspectives and issues for health behavior change interventions. Psychol Heal. (2015) 30:35–51. 10.1080/08870446.2014.953530 25140439

[47] Gyllensten K Palmer S. Can coaching reduce workplace stress? A quasi-experimental study. Int J Evid Based Coach Mentor. (2005) 3:75–85. 33495254

[48] Sarrafzadegan N Baghaei A Sadri G Kelishadi R Malekafzali H Boshtam M . Isfahan healthy heart program: evaluation of comprehensive, community-based interventions for non-communicable disease prevention. Prev Control. (2006) 2:73–84. 10.1016/j.precon.2006.10.003

[49] Harris A Bradham D Baumgarten B Zuckerman I Fink J Perencevich E. The use and interpretation of quasi-experimental studies in infectious diseases. Clin Infect Dis. (2004) 38:6. 10.1086/420936 15156447

[50] Rossi PH Lipsey MW Freeman HE. Assessing Program Impact. Alternative designs. Evaluation A Systematic Approach. Thousand Oaks, CA: SAGE (2004).

[51] Schweizer ML Braun BI Milstone AM. Research methods in healthcare epidemiology and antimicrobial stewardship-quasi-experimental designs. Infect Control Hosp Epidemiol. (2016) 37:1135–40. 10.1017/ice.2016.117 27267457 PMC5036994

