# Supplementary material for: Evaluation of a Sexual Transmitted Infection Prevention Program Among University Students in Beira City Central Mozambique: A Study Protocol
Source: Front Reprod Health. 2021 Oct 28;3:745309. doi: 10.3389/frph.2021.745309 (PMC9580759; doi:10.3389/frph.2021.745309)
Supplement: Supplementary file 1 [file Data_Sheet_1.PDF]

Appendix 1**: Matrices of changes for target group in the "*Take to opportunity to listen and to be listened to*" programme evaluation research**

	**Behaviour:BO1. University students engage in protected sexual intercourse practices (i.e., consistently and correctly use condoms)**					
	**Personal determinants**					
**Performance Objectives (University students)**	**Knowledge**	**Risk perception**	**Attitude**	**Self-efficacy**	**Skills**	**Subjective norms**
**PO1.1.** Students decide to use condom as the best mean of protection to STI/HIV.	K1.1.a. Describe means of protection against STI/HIV acquisition.		A1.1a. Express positive feeling regarding benefits of preventing STI.	SE1.1.a. Express confidence in ability to prevent STI acquisition.		
	K1.1b. Restate that using condom during sexual intercourse reduce the risk of acquiring STIs including HIV.	RP1.1.a Recognize that carrying an STI increase in 1/3 the chance to contract HIV.	A1.1.b. Express positive feeling regarding benefits of using condom consistently and correctly to reduce the risk of acquiring STI/HIV.	SE1.1.b. Express confidence in ability to make the decision to use condoms.		SN1.1.b. Recognize that most of sexually active students who decide to use condoms are more likely to use continuously.

**Continuation**: Matrices of changes for target group in the "*Take to opportunity to listen and to be listened to*" programme evaluation research _ BO 1						
**PO1.2.** Students locate condoms display points at health centre, health provider office, restrooms or at chemistry.	K1.2.a. Name places to buy or obtain condoms.			SE1.2.a. Express confidence in ability to buy or obtain condoms.		SN1.2.a. Recognise that other students obtain condoms for free at health centre, health provider office or by at the chemistry.
				SE1.2.b. Express confidence in ability to deal with embarrassment when buying or obtaining condoms.	S1.2.b. Demonstrate ability to deal with embarrassment when buying or take condoms.	
**PO1.3.** Students negotiate the use of condom with partner stating mutual goals such as preventing STI/HIV transmission.	K1.3.a. Record the steps of successful negotiation .		A1.3.a. Express positive feeling regarding benefits of successful negotiation.	SE1.3.a. Express confidence in ability to negotiate the use of condom with partner.	S1.3.a. Demonstrate ability to negotiate the use of condom with partner.	SN1.3.a. Recognize that sexual active peer students negotiate the use of condom with their partner.

Continuation: Matrices of changes for target group in the “*Take to opportunity to listen and to be listened to*” programme evaluation research _ BO1						
**PO1.4**. Students actively listen to their partner's concerns to use condom as a pre-condition for sex.	K1.4.a. Define components of active listing to a partner.		A1.4. a. Express positive feeling regarding benefits of respect to a partner's concerns.	SE1.4.a. Express feeling confident in ability to listen actively a partner's concerns about pre-conditions for sex.	S1.4.a. Demonstrate ability to listen actively to partner's concerns about pre-conditions for sex.	SN1.4.a Recognize that sexually active students actively listen and understand their partner's concerns.
**PO1.5.** Students apply condom correctly during use by using new condom at each event of intercourse and following structures for use and disposal.	K1.5.a. Describe how to correctly apply a condom.	RP1.5.a. Discuss risk level of STI/HIV acquisition in unsafe sexual intercourse.	A1.5.a. Express positive feeling regarding advantages of using condom correctly and consistently.	SE1.5.a. Express felling confident in ability to evaluate personal risk to STI/HIV acquisition<br><br>SE1.5.b. Express felling confident in ability to use condom correctly and consistently.	S1.5.a. Demonstrate ability to perform their risk self-evaluation.<br><br>S1.5.b. Demonstrate ability to use condom correctly.	SN1.5.a. Recognize that sexually active students use condom correctly and consistently.

	**BO2. University students regularly use STI/HIV screening services**					
	**Personal determinants**					
**Performance objectives (University students)**	**Knowledge**	**Risk Perception**	**Attitude**	**Self-efficacy**	**Skills**	**Subjective norms**
**PO2.1.** Students locate STI/HIV screening service office at the faculty or health centre.	K2.1.a. Recognise symptoms of possible STI infection.	RP2.1.a. Describe some conditions need to develop STI/HIV infection.	A2.1.a. Express positive feeling regarding benefits of STI/HIV adequate treatment.			
	K2.1.b.List steps to follow in case suspect having any STI/HIV.	RP2.1.b. Discuss complications of untreated STI.				
	K2.1.c. Recognize that attending STI/HIV screening test reduce spreading of STIs including HIV.		A2.1.b. Express positive feeling regarding benefits of using STI/HIV screening services.			

Continuation: Matrices of changes for target group in the "*Take to opportunity to listen and to be listened to*" programme evaluation research _ BO2

PO2.2. Students setup a calendar for regular visit to the health advisor for counselling and testing to STI/HIV.	K2.2.a. State the recommended time interval to regularly check or screening for STI/HIV.	RP2.2.a. Describe burden of HIV in Mozambique and its epidemic course.<br><br>RP2.2.b. Recognize that they are at risk for STIs/HIV acquisition.	A2.2.a. Express positive feeling about benefits of setting up a personal calendar for STI/HIV screening.	SE2.2.a. Express confidence in ability to setup a calendar to visit a health provider services for STI/HIV counselling and testing.	S2.2.a. Demonstrate ability to plan their time for STI/HIV check-up.	SN2.2.a. Recognise that they know other student who follows a personal calendary for STI/HIV screening.
PO2.3. Students visit a health advisor and present their concerns about STI/HIV.	K2.3.a. Describe clinical aspect related to STI/HIV infection.<br><br>K2.3.b. Discuss stages of STI/HIV infection development.	RP2.3.a. Recognize that they should get help from a health advisor very soon.	A2.3.a. Express positive feeling regarding benefits of getting help from health advisor soon.	SE2.3.a. Express feeling confident to tell their concerns about STI/HIV to the health advisor.	S2.3.b. Demonstrate ability to describe their concerns and/or symptoms.	SN2.3.a. Describe that they see other students going to the health advisor.
	K2.3.c. Describe myths and fears about STI/HIV testing.			SE2.3.b. Express confidence in ability to deal with fear of being judged (e.g. cheating) or having to inform current sexual partner.	S2.3.a. Demonstrate ability to use recommended words when talking about HIV concerns to avoid stigma and discrimination.	

**Continuation**: Matrices of changes for target group in the "*Take to opportunity to listen and to be listened to*" programme evaluation research _ BO2						
PO2.4. Students agree and accept testing results and inform their sexual partner.	K2.4.a. Describe the STI/HIV testing process.	RP2.4.a. Discuss consequences of delay learning about their STI/HIV condition.	A2.4.a. Express positive feeling regarding benefits of learning about their test results.	SE2.4.a. Express confidence in ability to deal with test results emotional reactions		
PO2.5. Students with positive test results go to the referred health centre for treatment and follow up.	K2.5.a. Describe the effect of medicines used to treat STI including anti-retroviral medicines.	RP2.5.a. Discuss consequences of delay treatment of STI/HIV	A2.5.a. Express positive felling regarding advantages of treatment and follow up for STI/HIV.	SE2.5.a. Express confidence in ability to go to the health centre for treatment and follow up if needed.		SN2.5.a. Recognize that other people with STI/HIV go to the health centre for treatment and follow up.
PO2.6 Students follow their calendar continuously.			A2.6.a. Express positive felling regarding regular visit to the health advisor for STI/HIV counselling and testing.	SE2.7.a. Express confidence in ability to follow their calendar continuously.	S2.7.a. Demonstrate ability to update their calendar.	SN2.7.a. Recognize that other students follow their calendar continuously.
