# Supplementary material for: Evaluation of a Sexual Transmitted Infection Prevention Program Among University Students in Beira City Central Mozambique: A Study Protocol
Source: Front Reprod Health. 2021 Oct 28;3:745309. doi: 10.3389/frph.2021.745309 (PMC9580759; doi:10.3389/frph.2021.745309)
Supplement: Supplementary file 2 [file Data_Sheet_2.PDF]

Appendix 2**: Methods and application for all components**

Methods are a theoretical-and evidence-based techniques and ways to change a determinant, and practical application is the idea about how to deliver a method to the target group or intermediary.

**Module/lesson**	**Assignment/Content**	**PO. Determinants and CO (examples)**	**Method**	**Application**	**Material/Vehicle**
Unprotected sex and Sexual Transmitted Infection (STI) including HIV.	Consequences of unprotected sex	PO1.1 Make the decision to use condom.<br>***Determinants***: Knowledge, risk perception, attitude and self-efficacy.<br><br>***CO***: Describe means of protection to STI/HIV and consequences of acquiring any STI including HIV.	Active learning or Elaboration (Knowledge, risk perception and attitude).<br><br>Reinforcement (knowledge, attitude, self-efficacy and risk perception).	The students solve the exercise about the wall of risks in unprotected sex, infidelity, and the door of opportunities, in groups. They list consequences of unprotected sex and infidelity and identify possible means of protection to STI/HIV acquisition.<br>The teacher discusses with students the general pros and cons of unprotected sexual intercourse and infidelity. Provide the list of consequences, emphasizing that the consequences might affect their life for long time. Thus, the students will realize that using condom is the most effective mean of protection to STI/HIV acquisition.<br><br>The students watch a theatre scene (5 to 10 min), about consequences of unprotected sexual intercourse.<br>At the end Volunteer Students (VE) make questions to the participant to assess what they have learned from the scene.<br>The participants give their opinion regarding need to decide to use condom in sexual intercourse.<br>VE give feedback and a summary addressing benefits of using condom for their protection to STI/HIV.	Students’ and teachers’ handbooks with background information and instructions.

Continuation: **Methods and application for all components**					
	Location and negotiation of condom use.	***PO1.2.*** Locate condom display point.<br>***Determinants:*** Knowledge, self-efficacy and Subjective norms.<br>***CO***: Locate place with condom, express confidence to obtain condom and recognise that other students obtain condoms from the same places.<br><br>***PO1.3***. Negotiate the use of condom with sexual partner.<br><br>***Determinants:*** Knowledge, attitude, self-efficacy, skills and subjective norm.<br><br>CO***:*** Record the steps and benefits of successful negotiation.	Facilitation (all determinants).<br><br>Planning copping response (self-efficacy and skills).<br><br>Shifting focus (Subjective norms).<br><br>Anticipated regret (attitude).	All sexually active students obtain condom from the health advisor office, health centres or buy it from the pharmacy. But the health advisor and manager of health centres will ensure that condoms are available at any time for clients.<br><br>The students and the health advisor will define causes of non-success (e.g., barrier) and formulate actions to copy the barrier of non-success in negotiation for condom use.<br><br>The students will be advised by the health advisor to negotiate condom use stating that it is to prevent pregnancy.<br><br>Students will watch a short theatre scene addressing how a couple would feel after having unprotected sex as a result of failing negotiation to use condom. The participants will discuss about the scene. And VE will provide feedback with clear steps of negotiation.	STI counselling and testing guideline of Mozambique.<br><br>Video ply.

Continuation: **Methods and application for all components**					
		PO1.4. students listen actively to their partners' concerns.<br><br>***Determinants:*** Knowledge, attitude, self-efficacy, skills and Subjective norms.<br><br>***CO:*** Demonstrate knowledge and ability to actively listen to partner's concerns for successful negotiation of condom use.	Discussion (all determinants).<br><br>Guided practice (Skill and self-efficacy).	After a short video ply, the students with the volunteer will discuss what they learned from the video about negotiation and active listening to a partner, then the VE will provide specifics tips of actively listening.<br><br>Students will watch a video play simulating a conversation with actively listening shape of a couple. Then students will be asked to do so in pairs. The VE will provide comments on pairs performance, emphasizing what was well done.	Video ply.

Continuation: **Methods and application for all components**					
	Use condom correctly and consistently.	***PO1.5.*** Students correctly apply condom during use and use it consistently.<br><br>***Determinants:*** Knowledge, risk perception, attitude, self-efficacy, skills and subjective norms.<br><br>CO: Express confidence in ability to evaluate risk to STI/HIV in unprotected sex.<br>Demonstrate ability to use condom correctly	Active learning (all determinants).<br><br>Personalise risk (risk perception).<br><br>Elaboration (Knowledge).<br><br>Reinforcement (all determinants).<br><br>Using imagery (Skills)	Students will determine the risk levels (high, medium, low, null) of STI/HIV transmission in different scenarios of sexual practices, though solving an exercise in their handbook, after receiving instructions from teacher.<br>Teacher will ask some students to present their responses. At the and the teacher will provide feedback and the correct answers from his/her handbook.<br>Teacher will ask students to be in different unsafe situation and indicate their risk level by choosing one of the three possible answers (A,B,C) in 5 minutes. One student will count how many students are in each answer (A,B,C).<br>Then the teacher will provide the response (A = High risk plyer; B = Medium risk plyer; C = safe plyer).<br>In a meeting, students are asked to write on the whiteboard or flip paper, one answer to the question: what does it means use condom correctly and consistently?<br>After all ideas are written, the VE will give feedback and summarise the answer with participants, setting up a list of actions that describe correctly and consistently use of condom.<br>Health advisors use pen mould and condom to demonstrate how to use it to the clients. The client practices the procedure.	Student and teacher handbooks with background information and instructions.<br><br>Student and teacher handbooks with background information and instructions<br><br>Paper print and with board<br><br>STI counselling and testing guideline of Mozambique.

Continuation: **Methods and application for all components**					
STI/HIV counselling and testing.	Progression of HIV Infection.	***PO2.1***. students locate STI/HIV screening services at the faculty.<br>***Determinants:*** Knowledge, risk perception and attitude.<br>***CO:*** Describe symptoms of STI and steps to follow for help.<br>Discuss complications of untreated STI and feeling positive about adequate treatment of STI/HIV.	Active learning (all determinants).<br><br>Facilitation (all determinants)	Students in group give answers to 5 questions addressing STI symptoms and signals, consequences of untreated STI in male and female, STI that can be cured and what a person must do in case discover that has an STI. Students will find some answers from their handbook information background.<br>Teachers provide feedback and emphasizes that STI increase the chance to contract HIV, so they should get help from the health advisor as soon as possible.<br>All students locate the health advisor office at the faculty in case they have any complain or concern related to their health.	Student and teacher handbooks with background information and instructions.
		PO2.2. Student setup a calendar for regular visit to the health advisor for counselling and testing to STI/HIV.<br>***Determinants:*** Knowledge, Risk perception, attitude, skills and subjective norms.<br>***CO:*** State recommended time for regular screening; Recognise that they are at risk for STI/HIV infection; express positive feeling and confidence to setup a calendar and recognise that other youth do so.	Personalize risk (all determinants).<br><br>Self-monitoring of behaviour (Skills and self-efficacy)	Students read their handbook and learn that they are at the high-risk age group for STI/HIV acquisition. Also receive personal risk information on their lack to visit any STI/HIV screening and testing services regularly from health advisor counselling. Students are recommended to read and interpret the map showing HIV burden in Mozambique in their handbook. Students give answers to the questions. Teachers give an overview regarding the burden of HIV in Mozambique.<br>Students will keep a calendar on their check-up adherence to regular screening for STI/HIV, to identify why and when they missed the visit.	Student and teacher handbooks with background information and instructions<br><br>STI counselling and testing guideline of Mozambique

Continuation: **Methods and application for all components**					
		PO2.3. Students visit a health advisor and present their concerns about SIT/HIV.<br><br>***Determinants:*** Knowledge, risk perception, attitude, Skills, and subjective norms<br>***CO:*** Describe clinical aspects, myths, and fear about STI/HIV; Recognise the need for help soon and express feeling benefits of that; Demonstrate ability to talk about STI/HIV and to use recommended words.	Active learning (all determinants).<br><br>Framing (risk perception).	Students will read the information background about stages of HIV infection (HIV infection, possible unspecific symptoms, asymptomatic stage, unspecific symptomatic stage, possible opportunistic diseases, and death) on their handbook; teacher will ask students to explain the stages of HIV infection from the graphic displayed on their handbook; The students will explain stages of infection development. Teacher will summarize the stages with class.<br><br>Students will be instructed to perform a theatre scene which address several scenarios in HIV testing context: (acceptance to HIV testing; needless to do test; fear to do test; abandonment during counselling; change theme of conversation and acting as senseless). The teacher will interrupt the scene after all scenarios, and guide students to analyse and discuss about different perceptions, myths and beliefs regarding HIV testing.<br>Finally, the teacher will summarise telling students that there are advantages if students accept HIV testing to know their state and list all possible myths and fears of persons regarding HIV testing.	Students' and teachers' handbook with background information and clear instructions to perform the activities.

Continuation: **Methods and application for all components**					
		PO2.4. Students agree and accept testing results.<br><br>***Determinants:*** Knowledge, risk perception, attitude, self- efficacy<br><br>**CO:** Describe STI/HIV test procedure; recognise consequences of delay testing; feeling positive regarding benefits of early STI/HIV diagnose and feeling confident to deal with test results.	Facilitation (all determinants).<br><br>Motivational interviewing (all determinants).<br><br>Arguments (all determinants).<br><br>Motivational interviewing (all determinants).<br><br>Personalised risk (risk perception).	All students with STI/HIV concerns will go to the health advisor office for counselling and testing, free of myths and fears at the faculty.<br>Students will receive from the health advisor counselling sessions (pre-test and during testing) in a collaborative goal-oriented style of communication, focusing more in the language of change in order to strengthen personal intention for commitment to deal with fear, myths and judgements from any source.<br>Students will attend lesson addressing HIV testing procedures, test result mining and benefits of learning about their results, in a lecture provided by teacher.<br>Students will receive from the health advisor counselling session (post-test) in a collaborative goal-oriented style of communication, focusing more on the language of change in order to strengthen personal intention for commitment to deal with emotional aspects according to their testing results.<br>Students will receive personal risk information according to their test results from the health advisor.	STI counselling and testing guideline of Mozambique.<br><br>Student and teacher handbooks with background information and instructions<br><br>STI counselling and testing guideline of Mozambique.

Continuation: **Methods and application for all components**					
Anti-retroviral treatment (ARV).	Criterions and effects of ARV treatment.	***PO2.5.*** Students with positive test results go to the indicated health centre for treatment and follow up.<br><br>***Determinants:*** Knowledge, risk perception, attitude, self-efficacy and subjective norms.<br><br>***CO:*** Describe the effect of medicines in treatment of STI/HIV; recognise benefits of early treatment; feeling positive and confident about early treatment of STI/HIV.	Active learning (all determinants)<br><br>Elaboration (knowledge)<br><br>Motivational interviewing (all determinants).<br><br>Scenario-based risk information (risk perception).	Students will respond to question: what they know about ARV treatment? When it must be initiated? And what is the function of ARV. The teacher will recommend students to read their handbook about the background information in regard.<br>Teacher will guide a discussion of students answers with class, at the and the teacher will summarise the content.<br><br>Students with positive test result will receive from the health advisor post-test counselling in a collaborative goal-oriented style of communication, focusing more on the language of change in order to strengthen personal intention for commitment to go to the health centre for treatment and follow up.<br>The students watch a theatre scene (5 min), about consequences of delayed treatment to STI/HIV.<br>At the end VE make questions to the participant to assess what they have learned from the scene.<br>The participants give their opinion on consequences of delay and about benefits of early treatment to STI/HIV.<br>VE give feedback and summary addressing benefits of early treatment to STI/HIV.	Student and teacher handbooks with background information and instructions<br><br>STI counselling and testing guideline of Mozambique.<br><br>Video tape

Continuation: **Methods and application for all components**					
	Calendar for STI/HIV screening.	***PO2.6.*** Students follow their calendar continuously.<br>***Determinants:*** Knowledge, attitude, self-efficacy, skills and subjective norms.<br><br>***CO:*** Express felling benefits and confident to follow their calendar STI/HIV screening.<br>Demonstrate ability to update their calendar, recognise that other students do so.	Motivational interviewing (all determinants).<br><br>Self-monitoring of behaviour (skills and self-efficacy).<br><br>Information about others' approval (social norms).	All clients will receive from the health advisor post-test counselling in a collaborative goal-oriented style of communication, focusing more on the language of change in order to strengthen personal intention for commitment to follow their calendar for STI/HIV screening continuously.<br><br>Students will keep a calendar on their check-up adherence to regular screening for STI/HIV, to identify why and when they missed the visit.<br><br>Students will be given data showing the percentage of students who follows their calendar of STI/HIV screening every three months.	STI counselling and testing guideline of Mozambique.<br><br>Graphics displayed in public places in all study settings.
