# Supplementary material for: Evaluation of a Sexual Transmitted Infection Prevention Program Among University Students in Beira City Central Mozambique: A Study Protocol
Source: Front Reprod Health. 2021 Oct 28;3:745309. doi: 10.3389/frph.2021.745309 (PMC9580759; doi:10.3389/frph.2021.745309)
Supplement: Supplementary file 3 [file Data_Sheet_3.PDF]

Evaluation of a sexual transmitted infection prevention program among university students in Beira city, Central Mozambique

Name of data collector: _________________________________ Date of filling: __/___/202__

Collection setting (university/faculty/class) ________/_______________________________ /________

Participant Identification Number ___/___/___ Number of visit: 0__ 1__ 2__

Section 1: Sociodemographic and living characteristics

QN.	Question	Code of possible answers	Pass
Q101	What is your sex?	Female 1<br>Male 2	
Q102	How old were you at your last birthday?	Years of old ☐☐	
Q103	What is your religion?	None 1<br>Catholic 2<br>Methodist 3<br>Anglican 4<br>Muslim 5<br>Hindu 6<br>Other ....7<br>(Specify)	→ Q105

Q104	How often do you attend religious activities?	Never 1<br>Almost never 2<br>Some days/occasionally 3<br>Almost everyday 4<br>Everyday 5	
Q105	**Now we invite you to answer some questions about your family!**<br>Is your father alive?	Yes 1<br>No 2	→ Q109
Q106	Does he live in the same household as you?	Yes 1<br>No 2	
Q107	Do you find it difficult to talk with your father about important thinks for you?	Very difficult 1<br>Difficult 2<br>Neutral 3<br>Easy 4<br>Very easy 5<br>Do not see him 6	→ Q109
Q108	Have you ever discussed sexuality-related matters with your father? If YES, how often.	Never 1<br>Rarely 2<br>Occasionally 3<br>A moderate amount 4<br>A great deal 5	

Q109	Is your mother alive?	Yes 1<br>No 2	 → Q201
Q110	Does she live in the same household as you?	Yes 1<br>No 2	
Q111	Do you find it difficult or easy to talk with your mother about important thinks for you?	Very difficult 1<br>Difficult 2<br>Neutral 3<br>Easy 4<br>Very easy 5<br>Do not see her 6	
Q112	Have you ever discussed sexuality-related matters with your mother? If YES, how often.	Never 1<br>Rarely 2<br>Occasionally 3<br>A moderate amount 4<br>A great deal 5	

Evaluation of a sexual transmitted infection prevention programme among university students in Beira city, Central Mozambique

Name of data collector: ________________________________ Date of filling: __/___/202__

Collection setting (university/faculty/class) ________/___________________________/__________

Participant code number ___/___/___ Visit number: 0 __ 1__ 2 ___

Section 2: Current or most recent heterosexual relationship

QN.	Question	Code of possible answer	Pass
Q201	Have you ever had a boy/girlfriend? By girl/boyfriend we mean someone to whom you were sexually or emotionally attracted and whom you are in love with.	Yes 1<br>No 2	→ Q214
Q202	How many girl/boy friends have you had?	Number ☐☐	
	Answer the following questions about current girl / boy friend		
Q203	How old is your current girl / boyfriend?	Age in years ☐☐	
Q204	When you become in love with your girl / boyfriend was she/he single, married, divorced or separated?	Single 1<br>Married 2<br>Divorce/Separate 3	

		Widow 4	
Q205	When you become in love with your girl / boyfriend was she/ he a full-time student, working or neither?	Full time student 1<br>Working 2<br>Neither 3	
Q206	How would you describe your relationship with your current lover? Is it a) casual friendship; b) a serious relationship; or c) an important that might lead to marriage?	(a) Casual 1<br>(b) Serious 2<br>(c) Important/might lead to marriage 3<br>(d) Engaged to be married 4<br>(e) The relationship ended 5	
Q207	How do you think your lover would describe her/his relationship to you?<br>a) as casual friendship; b) a serious relationship but with no intention of marriage; or c) an important that might lead to marriage?	(a) Casual 1<br>(b) Serious 2<br>(c) Important/might lead to marriage 3<br>(d) Engaged to be married 4	
Q208	Have you ever had sexual intercourse with your lover?	Yes 1<br>No 2	→ Q214
Q209	Remember about the last time you had sex with your lover, would you say:<br>a) I forced she/he to have intercourse against her/his will.<br>b) Persuaded she/he to have intercourse.<br>c) She/he persuaded me to have intercourse.<br>d) She/he forced me to have intercourse.<br>e) We were both equally willing.	a) I forced 1<br>b) I persuaded 2<br>c) She/he persuaded 3<br>d) She/he forced 4<br>e) Both Willing 5	

Q210	How old were you at the time you first had sex intercourse in your life?	Age in years ☐☐	
Q211	Were you ever concerned that you might catch HIV/AIDS or another STI/STD from your lover? If YES, very, or somewhat?	Very concerned 1<br>Somewhat concerned 2<br>Not concerned 3	
Q212	Were you able to do anything to reduce the risk of acquiring any infection?	Yes 1<br>No 2	
Q213	What did you do?	Use condom 1<br>Take medicine 2<br>Other 3<br>……………………………………………………<br>(Specify)	
	NOTE! Q214 and Q215 only for who did not engage in sexual activities yet!		
Q214	People may have many reasons for not having sexual intercourse. If that is your case, please tell us for each reason whether it applies to you or not:<br>a) I do not feel ready to have sex.<br>b) I have not had the opportunity.<br>c) I think sex before marriage is wrong.<br>d) I am afraid of getting pregnant.	Applies Doesn't apply Not sure<br>1 2 3<br>1 2 3<br>1 2 3<br>1 2 3	

	e) I am afraid of getting STI/STD or HIV/AIDS.	1 2 3	
Q214	**Now we would like to know about your future plan regarding sexual intercourse. Please indicate which of these statements best describe your plans:**<br><br>a) I plan to wait until marriage.<br>b) I plan until I am engaged to be married.<br>c) I plan to wait until I find someone I love.<br>d) I plan to have sexual intercourse when an opportunity comes along.	a) Marriage 1<br>b) Engaged 2<br>c) Lover 3<br>d) Opportunity 4	

Evaluation of a sexual transmitted infection prevention programme among university students in Beira city, Central Mozambique

Name of data collector: ________________________________ Date of filling: __/___/202__

Collection setting (university/faculty/class) __________/__________/__________________________

Participant code number ___/___/___ Visit number: 0 ___ 1 ___2 ___

Section 3: BO1. University students engage in safe sexual intercourse practices (i.e., consistently and correctly use condoms)

QN.	Question	Code of possible answer
	**Now we are inviting you to answer some question regarding your knowledge about condom use.**	
Q.K1.1.a	What of these are consequences of not using condom at any sexual intercourse? (**circle all possible answers**).	Acquire tuberculosis 1<br>Acquire Syphilis 2<br>Acquire gonorrhoea 3<br>Acquire HIV 4<br>Acquire any STI/STD 5<br>Become pregnant/Make pregnant your girlfriend 6
Q.K1.1.b	Using condom during sexual intercourse eliminate completely the risk of acquiring STI/STD including HIV.	Yes 1<br>No 2<br>Do not know 3

Q.K1.2.a	Where do think that you can buy or obtain condom? (**select all possible place).**	At the health centre 1<br>At supermarket 2<br>In the health advisor office 3<br>At informal market 4<br>At the chemistry 5<br>Other 6<br>Specify…………………………………………
Q.K1.3.a	Where could you keep condoms that you consider private and effective for you to use in case you have sexual intercourse? (**select all possible place).**	In my wallet 1<br>In my school bag 2<br>In my pocket 3<br>In my purse 4<br>In my bookshelves 5<br>Under my pillow 6<br>Other places 7<br>Specify…………………………………….
Q.K1.3.b	Condoms have lifetime, but it is not necessary to check its validity before obtaining or use, because the provider does it for the clients.	Yes 1<br>No 2<br>Don't know 3
Q.K1.4.a	Which of these statements are steps of negotiation for condom use?<br>a) Ask your partner if he/she willing to use condom.<br>b) Brainstorm about benefits of using condom.	a) Ask your partner 1<br>b) Discuss benefits 2

	c) Persuasive argument and actively listening.<br>d) Making consensus and getting agreement.<br>e) Keep your opinion fixed.	c) Persuasive and listen 3<br>d) Consensus/agreement 4<br>e) Fixed opinion 5<br>f) No idea 6
Q.K1.6.a	Actively listen consist of:<br><br>***Select all possible characteristics.***	Hearing without replay to 1<br>Understand the message 2<br>Serious facial expression 3<br>Eye contact 4<br>No idea 5
Q.K1.7.a	Condom can be evaluated by checking the presence of estrange particles on it.	Yes 1<br>No 2<br>Do not know 3
Q.K1.7.b	Which of the statements correspond to correct use of condom?<br><br>a) Use a new condom for every act of vaginal, anal and oral sex.<br>b) Before genital contact, put the condom on the tip of the erect penis with the rolled side out.<br>c) If the condom does not have a reservoir tip, pinch the tip enough to leave a half-inch space for semen to collect.<br>d) After ejaculation and before the penis gets soft, hold the edge of the condom and carefully withdraw.<br>e) Wrap the condom in a tissue and throw it in the trash where others will not handle it.	a) New in every sex act 1<br>b) Condom on erect penis 2<br>c) Ensure space for semen 3<br>d) Remove after ejaculation 4<br>e) Safely throw in a trash 5<br>f) Never interrupt sex act 6

	f) If you feel the condom break at any point during sex act, continue normally.<br>g) Water-based lubricants might be used if needed	g) Use water-based lubricants 7<br>h) No idea 8
Q.K1.7.c	A condom must be removed immediately after ejaculation and disposed in the kitchen waste bin.	Yes 1<br>No 2<br>Do not know 3
	**Now we are inviting you to answer some question regarding your attitudes about condom use.**<br>Instructions:<br>Many questions in this section use a ranting scale with 7 levels (good…Bad or Agree…Disagree, or likely…. unlikely). You are requested to circle the number which better describe your opinion.	Example:<br>7 = Agree completely<br>6 = Quite agree<br>5 = Agree slightly<br>4 = Neither agree nor disagree<br>3 = Disagree slightly<br>2 = Quite disagree<br>1= Disagree completely
Q.A1.1.a	Using condom at sexual intercourse reduce pleasure.<br>*Please, cycle the number which describe better your opinion!*	Disagree……………………… Agree<br>1 2 3 4 5 6 7
Q.A1.1.b	Using condom consistently and correctly is beneficial and reduce the risk of acquiring STI and/or HIV.<br>*Please, cycle the number which describe better your opinion!*	Disagree…………………… Agree<br>1 2 3 4 5 6 7

Q.A1.3.a	Keeping condom with you in a private and effective place is beneficial.<br>*Please, cycle the number which describe better your opinion!*	Disagree.............................. Agree<br>1 2 3 4 5 6 7
Q.A1.3.b	keeping condoms with you within the validity time has benefits.<br>*Please, cycle the number which describe better your opinion!*	Disagree.............................. Agree<br>1 2 3 4 5 6 7
Q.A1.4.a	Successful negotiation for using condom in a sex act has benefits.<br>*Please, cycle the number which describe better your opinion!*	Disagree..............................Agree<br>1 2 3 4 5 6 7
Q.A1.5.a	State pre-condition and pre-requisite for sexual intercourse has benefits.<br>*Please, cycle the number which describe better your opinion!*	Disagree.............................. Agree<br>1 2 3 4 5 6 7
Q.A1.6.a	Respect to a partner's concerns regarding sex act is important.<br>*Please, cycle the number which describe better your opinion!*	Disagree.............................. Agree<br>1 2 3 4 5 6 7
Q.A1.7.a	Using a new condom at each sex event has benefits.<br>*Please, cycle the number which describe better your opinion!*	Disagree..............................Agree<br>1 2 3 4 5 6 7
Q.A1.7.b	Using condom correctly has benefits.<br>*Please, cycle the number which describe better your opinion!*	Disagree..............................Agree<br>1 2 3 4 5 6 7

Q.A1.7.c	Safe removal and disposal of a condom is important.<br><br>*Please, cycle the number which describe better your opinion!*	Disagree………………………. Agree<br><br>1 2 3 4 5 6 7
	**Now we are inviting you to answer some question regarding your Self-efficacy about condom use**<br><br>Similar as the previous group of questions, *Please, cycle the number which describe better your opinion!*	Example:<br><br>7 = Extremely likely/agree<br><br>6 = Quite likely/agree<br><br>5 = Slightly likely/agree<br><br>4 = Neither likely nor unlikely<br><br>3= Slightly unlikely/disagree<br><br>2 = Quite unlikely/disagree<br><br>1 = Extremely unlikely/disagree
Q.SE1.1a	I am confident to make the decision to use condoms.<br><br>*Please, cycle the number which describe better your opinion!*	Unlikely…………………………Likely<br><br>1 2 3 4 5 6 7
QSE1.2.a	I am confident to buy or obtain condoms any display point.	Unlikely…………………………Likely<br><br>1 2 3 4 5 6 7
Q.SE1.2.b	I am confident to deal with embarrassment when buying or take condoms.	Unlikely…………………………Likely<br><br>1 2 3 4 5 6 7
Q.SE1.3.a	I am confident to carry condoms discreetly.	Unlikely…………………………Likely<br><br>1 2 3 4 5 6 7

Q.SE1.4.a	To negotiate contraceptive use with a partner is difficult.	Unlikely…………………………Likely<br>1 2 3 4 5 6 7
Q.SE1.5.a	I am confident to convince a partner to use condom as a pre-requisite for sex intercourse.	Unlikely…………………………Likely<br>1 2 3 4 5 6 7
Q.SE1.5.b	I am confident to delay sex if negotiation fails.	Unlikely…………………………Likely<br>1 2 3 4 5 6 7
Q.SE1.6.a	I am confident to listen actively a partner's concerns about pre-conditions for sex intercourse.	Unlikely…………………………Likely<br>1 2 3 4 5 6 7
Q.SE1.7.a	I am confident to use condom correctly.	Unlikely…………………………Likely<br>1 2 3 4 5 6 7
Q.SE1.7.b	I am confident to removal and disposal condom after use.	Unlikely…………………………Likely<br>1 2 3 4 5 6 7
	**Now we are inviting you to answer some question regarding Subjective norms about condom use**	
Q.SN1.1.a	Most sexually active students think that it is important to use condom.	Disagree……………………….Agree<br>1 2 3 4 5 6 7
Q.SN.1.1.b	Most of sexually active students who decide to use condoms are more likely to use continuously.	Unlikely………………………… Likely<br>1 2 3 4 5 6 7
Q.SN1.2.a	Other students obtain condoms for free at health centre, health provider office or by at the chemistry.	Disagree………………………. Agree<br>1 2 3 4 5 6 7

Q.SN1.3.a	Most students carry condoms in a private place to easily access.	Disagree…………………………Agree<br>1 2 3 4 5 6 7
Q.SN1.4.a	Sexually active peer students negotiate the use of condom with their partner.	Disagree………………………… Agree<br>1 2 3 4 5 6 7
Q.SN1.5.a	Sexually active peer students delay sex when negotiation fails.	Unlikely……………………………. Likely<br>1 2 3 4 5 6 7
Q.SN1.6.a	Sexually active students actively listen and understand their partner's concerns.	Unlikely……………………………. Likely<br>1 2 3 4 5 6 7
Q.SN1.7.a	Sexually active students use condom correctly.	Disagree……………………………Agree<br>1 2 3 4 5 6 7

Evaluation of a sexual transmitted infection prevention programme among university students in Beira city, Central Mozambique

Name of data collector: _________________________________ Date of filling: __/___/202__

Collection setting (university/faculty/class) __________/__________/__________________________

Participant code number ___/___/___ Visit number: 0 __ 1 ___ 2 ___

Section 4: BO2. University students regularly use STI/HIV screening services

QN.	Question	Code of possible answer
	**Now we are inviting you to answer some question regarding your knowledge about use of STI/HIV screening services.**	
Q.K2.1.a	What of these are consequences of not using STI/HIV screening services? (***circle all possible answers).***	Transmission of STI/HIV 1<br>Evolution to chronic stage 2<br>Acquire HIV 3<br>Delay treatment 4<br>Hospitalization 5
Q.K2.1.b	Do you agree that using STI/HIV screening services reduce spreading of STIs including HIV.	Strongly disagree 1<br>Disagree 2<br>Neither agree nor disagree 3<br>Agree 4

		Strongly agree 5
Q.K2.2a	Which of the following time interval is recommended for regular screening of STI/HIV?	Once a year 1<br>Every 6 months 2<br>Every 3 months 3<br>Every month 4<br>Whenever you have concerns 5
Q.RP2.3.a	Are you aware about your risk for STI/HIV acquisition?	Not at all aware 1<br>Slightly aware 2<br>Somewhat aware 3<br>Aware 4<br>Extremely aware 5
Q.RP2.3.b	Do you agree that you should get help from a health advisor very soon for STI/HIV concerns?	Strongly disagree 1<br>Disagree 2<br>Neither agree nor disagree 3<br>Agree 4<br>Strongly agree 5
Q.K2.5.a	If your HIV test result is positive, how many lines the rapid test device shows?	None 1<br>Two, but one-line pale 2<br>Two lines pales 3<br>Two lines distinct 4<br>Don’t know 5
	**Now we are inviting you to answer some question concerning your**	

	**attitude regarding use of STI/HIV screening services.**<br>*Instructions:*<br>Many questions in this section use a ranting scale with 7 levels (Good….Bad, or Agree…Disagree, or Likely…. Unlikely). You are requested to circle the number which best describe your opinion.	**Example:**<br>7 = Completely agree<br>6 = Quite agree<br>5 = Slightly agree<br>4 = Neither agree nor disagree<br>3 = Slightly disagree<br>2= Quite disagree<br>1 = Completely disagree
Q.A2.1.a	Using STI/HIV screening services is pleasant.<br>*Please cycle the number which describe better your opinion!*	Disagree……………………………. Agree<br>1 2 3 4 5 6 7
Q.A2.2.a	Setting up a personal calendar for STI/HIV screening visit has benefits.<br>*Please cycle the number which describe better your opinion!*	Disagree……………………………. Agree<br>1 2 3 4 5 6 7
Q.A2.3.a	Getting help from health advisor about STI/HIV concerns is beneficial.<br>*Please cycle the number which describe better your opinion!*	Disagree……………………………. Agree<br>1 2 3 4 5 6 7
Q.A.2.3.b	Getting help from health advisors soon is beneficial.<br>*Please cycle the number which describe better your opinion!*	Disagree…………………………… Agree<br>1 2 3 4 5 6 7

Q.A2.4.a	Learning about your results regarding STI/HIV has benefits.<br><br>*Please, cycle the number which describe better your opinion!*	Disagree……………………… Agree<br><br>1 2 3 4 5 6 7
Q.A2.5.a	Accepting test results about your STI/HIV state is beneficial<br><br>*Please cycle the number which describe better your opinion!*	Disagree……………………… Agree<br><br>1 2 3 4 5 6 7
Q.A2.6.a	Going to the health centre for treatment and follow up to STI/HIV has benefits.<br><br>*Please, cycle the number which describe better your opinion!*	Disagree……………………….. Agree<br><br>1 2 3 4 5 6 7
Q.A2.7.a	Regular visit to the health advisor for STI/HIV counselling and testing has benefits.<br><br>*Please, cycle the number which describe better your opinion!*	Disagree……………………… Agree<br><br>1 2 3 4 5 6 7
	**Now we are inviting you to answer some question concerning your self-efficacy about use of STI/HIV screening services.**<br><br>Similar as the previous group of questions, *Please, cycle the number which describe better your opinion!*	Example:<br><br>7 = Extremely likely/agree<br><br>6 = Quite likely/agree<br><br>5 = Slightly likely/agree<br><br>4 = Neither likely nor unlikely<br><br>3 = Slightly unlikely/disagree<br><br>2 = Quite unlikely/disagree<br><br>1= Extremely unlikely/disagree

Q.SE2.2.a	I am able to setup a calendar to visit a health advisor services for STI/HIV counselling and testing.	Unlikely................................ Likely<br>1 2 3 4 5 6 7
Q.SE2.3.a	I am confident to tell my concerns about STI/HIV to the health provider.	Unlikely.................................. Likely<br>1 2 3 4 5 6 7
Q.SE2.3.b1	I am confident to deal with fear of being judged (eg. cheating).	Unlikely.................................. Likely<br>1 2 3 4 5 6 7
Q.SE2.3.b2	I am confident to inform my current sexual partner.	Unlikely.................................. Likely<br>1 2 3 4 5 6 7
Q.SE2.4.a	I am confident to pick up results from to the health advisor office.	Unlikely.................................. Likely<br>1 2 3 4 5 6 7
Q.SE2.5.a	I am confident to deal with having STI/HIV infection.	Unlikely.................................. Likely<br>1 2 3 4 5 6 7
Q.SE2.5.b	I am confident to deal with emotional reactions about the results.	Unlikely.................................. Likely<br>1 2 3 4 5 6 7
Q.SE2.6.a	I am confident to go to the health centre for treatment and follow up if needed.	Unlikely.................................. Likely<br>1 2 3 4 5 6 7
Q.SE2.7.a	I am confident to follow my calendar continuously.	Unlikely.................................. Likely<br>1 2 3 4 5 6 7
	**Now we are inviting you to answer some question regarding subjective norms about use of STI/HIV testing services.**	

	Similar as the previous group of questions; *Please, cycle the number which describe better your opinion!*	
Q.SN2.2.a	Other students like me are following their personal plan for STI/HIV screening.	Disagree……………………………. Agree<br>1 2 3 4 5 6 7
Q.SN2.3.a	Other students like me usually go to the health provider for STI/HIV complains.	Unlikely……………………………. Likely<br>1 2 3 4 5 6 7
Q.SN2.6.a	Other people with STI/HIV go to the health centre for treatment and follow up.	Disagree……………………………. Agree<br>1 2 3 4 5 6 7
Q.SN2.7.a	Other students will follow their calendar continuously.	Unlikely……………………………… Likely<br>1 2 3 4 5 6 7
Q.U2.1	Have ever visited a VCT?	Yes 1<br>No 2<br>Do not remember 3
Q.U2.2	When was the last time you visited a VCT?	1 to 4 weeks ago 1<br>1 to 3 months ago 2<br>4 to 6 months ago 3<br>A year ago 4<br>Do not remember 5
