# Supplementary material for: Evaluation of a Sexual Transmitted Infection Prevention Program Among University Students in Beira City Central Mozambique: A Study Protocol
Source: Front Reprod Health. 2021 Oct 28;3:745309. doi: 10.3389/frph.2021.745309 (PMC9580759; doi:10.3389/frph.2021.745309)
Supplement: Supplementary file 4 [file Data_Sheet_4.PDF]

Appendix 4a

Interview guide to activists

Name of data collector: ________________________________ Date of filling: __/___/202__

Collection setting (university/faculty/class) ________/______________________________ /________

Participant Identification Number ______________

QN.	Question	Code of possible answer	
VSO101	What is your sex?	Female<br>Male	1<br>2
VEO102	How old were you at your last birthday?	Years old ☐☐	
VSO103	How did you engage to the group?<br><br>***Please select only one option!***	My friend invited me to join<br>My teacher advised me to join<br>One of the activists invited me to join<br>I joint voluntarily<br>None of the above<br>Specify: _________________________	1<br>2<br>3<br>4<br>5
VSO104	To what extend do you think your engagement in sensitizing activities is important to yourself?<br>***Select all possible answers!***	Not at all important<br>Low important<br>Slightly important<br>Neutral<br>Moderately important<br>Very important<br>Extremely important	1<br>2<br>3<br>4<br>5<br>6<br>7
Please, can you explain why?			

VSO105	To what extend do you think your engagement in sensitizing activities is important or useful to your target group? ***Select all possible answers!***	Not at all important<br>Low important<br>Slightly important<br>Neutral<br>Moderately important<br>Very important<br>Extremely important	1<br>2<br>3<br>4<br>5<br>6<br>7
Please, explain why?			
VSO106	Do you think awareness activities about STI/HIV prevention are priority to first year students at university?	Not at all a priority<br>Low priority<br>Slightly priority<br>Neutral<br>Moderately priority<br>High priority<br>Essential priority	1<br>2<br>3<br>4<br>5<br>6<br>7
Please, explain why?			
VSO107	To what extend are you concerned with STI/HIV prevention among university students?	Not at all concerned<br>Low concerned<br>Slightly concerned<br>Neutral<br>Moderately concerned<br>Very concerned<br>Extremely concerned	1<br>2<br>3<br>4<br>5<br>6<br>7

Please, explain why?		
VSO108	To what extend do you think improvement of the program is needed?	Not at all needed 1<br>Low needed 2<br>Slightly needed 3<br>Neutral 4<br>Moderately needed 5<br>Very needed 6<br>Extremely needed 7
Please, tell me what do you think can be done to improve the program?		

Appendix 4b

Interview guide to Teachers

Name of data collector: _________________________________ Date of filling: __/___/202__

Collection setting (university/faculty) ________________________________ /_________________

Participant Identification Number _______________

QN.	Question	Code of possible answer	
LST101	What is your sex?	Female<br>Male	1<br>2
LST102	How old were you at your last birthday?	Years old ☐☐	
LST103	What is your basic training area?<br><br>***Please select only one option!***	Physician<br>Nurse<br>Lab technician<br>Psychology/Clinical Psychology<br>None of the above<br>Specify: _________________________	1<br>2<br>3<br>4<br>5
LST104: How did you engage in teaching life skills subject?			
LST105: How long have you been teaching life skill at university? (Number of year working as a life skills teacher) -------------			

LST106: Can you describe your experience with sexual and reproductive health contents?<br>Please describe your experience considering trainings, skills, motivation….
LST107: How do you describe the attendance of students to life skills lessons?

LST108: How do you describe the participation of students to life skills lessons?
LST109: Have you used other material to teach life skills along with program material? What for example?

Appendix 4c

Interview guide to health advisor

Name of data collector: _________________________________ Date of filling: __/___/202__

Collection setting (university/faculty)______________________________

Participant Identification Number _______________

QN.	Question	Code of possible answer
HAO101	What is your sex?	Female 1<br>Male 2
HAO102	How old were you at your last birth day?	Years old ☐☐
HAO103	What is your basic training area?<br><br>***Please select only one option!***	Community advisor 1<br>Health advisor 2<br>Nurse 3<br>Lab technician 4<br>Psychology 5<br>None of the above 6<br>Specify: _________________________
HAO104: How long have you been working as a health advisor? (Number of year working as a health advisor) ------------		
HAO105: How do you describe your experience with counselling and testing service provision? Please describe your experience considering trainings, skills, motivation….		
HAO106: What do you think about utiility of your work/activities for students?		

HAO107: What sort of problems students usually report when they visit your office?
HAO108: What are the main challenges in the implementation of the program?
HAO109: If you were asked to improve the program, what would you suggest in order to improve your participation as a health advisor?

Appendix 4d

Interview guide to program user

Name of data collector: ________________________________ Date of filling: __/___/202__

Collection setting (university/faculty/class) ________/______________________________ /________

Participant Identification Number ____________

Section 1: Life skills lessons process evaluation

QN.	Question	Code of possible answer
LPE101	What is your sex?	Female 1<br>Male 2
LPE102	How old were you at your last birthday?	Years old ☐☐
LPE103	How often do you attend life skills lessons?<br><br>***Please select only one option!***	Never 1<br>Rarely, in less than 10% of the time that I could attend 2<br>Occasionally, in about 30% of the time that I could attend 3<br>Sometime, in about 50% of the time that I could attend 4<br>Frequently, in about 70% of the time that I could attend 5<br>Usually, in about 90% of the time that I could attend 6<br>Every scheduled times 7
LPE104	Have you ever had the opportunity to discuss (solve the exercises) the topic individually or in group during life skills lessons?<br><br>***Please select only one option!***	Never have 1<br>Almost never have 2<br>Sometimes/occasionally 3<br>Almost every lesson 4<br>Every lesson 5
LPE105	When did you discuss or solve the exercise?<br>***Please select only one option!***	Never discuss 1<br>After lecture presentation 2<br>During lecture presentation 3<br>Before lecture presentation 4

LPE106	Does the teacher provide a summary of the topic?<br>***Please select only one option!***	Never provide 1<br>Almost never provide 2<br>Sometimes/occasionally 3<br>Almost every lesson 4<br>Every lesson 5
LPE107	When the teacher provides summary of the topic?<br><br>***Please select only one option!***	Never provide 1<br>Do not remember 2<br>At the beginning of the lecture 3<br>During the lecture 4<br>After discussion with students 5<br>At the end of the lecture 6

LPE108: What do you think about life skills lessons, special the lectures that address STI/HIV prevention at university? Please, feel free to express your personal feeling!

Section 2: Sensitising activities process evaluation

QN.	Question	Code of possible answer
SPE101	What is your sex?	Female 1<br>Male 2
SPE102	How old were you at your last birthday?	Years old ☐☐
SPE103	How often do you attend meetings with volunteer students (activists)?<br><br>***Please select only one option!***	Never 1<br>Almost never 2<br>Occasionally/Sometimes 3<br>Almost every time 4<br>Every time 5
SPE104	What happed during the meetings?<br>***Select all possible answers!***	A group of activists presented a theatre scene 1<br>A video ply was presented 2<br>The activists asked some question at the end of the presentation 3<br>The participants discussed or answered the questions 4<br>The activists provided a summary addressing benefits of performing a specific behaviour toward STI/HIV prevention and screening 5<br>Other 6<br>(Specify) ______________________________<br>____

SPE105: What do you think about awareness activities regarding STI/HIV prevention at university? Please, feel free to express your personal feeling!

Section 3: Counselling and testing process evaluation

**QN.**	**Question**	**Code of possible answer**
TPE101	What is your sex?	Female 1<br>Male 2
TPE102	How old were you at your last birthday?	Years old ☐☐
TPE103	How often do you visit the health advisor office?<br><br>***Please select only one option!***	Never 1<br>Every once a year 2<br>Every twice a year 3<br>Every three times a year 4<br>More four times a year 5<br>More than four times a year 6
TPE104	What do you usually discuss with the health advisor?<br>***Select all possible answers!***	Negotiation of condom use 1<br>Location of condom display 2<br>Use of condom correctly and consistently 3<br>STI/HIV testing procedures 4<br>STI/HIV testing results interpretation 5<br>Calendar for STI/HIV screening 6<br>Other 7<br>Specify) ________________________________<br>____
TPE105	During your visits to the health advisor, for counselling and testing purpose, are you responsible for the conversation?	Not at all responsible 1<br>Somewhat responsible 2<br>Mostly responsible 3<br>Completely responsible 4
TPE106	During post-test counselling would you consider the process as collaborative?	Would not consider 1<br>Might or might not consider 2<br>Definitely consider 3
	During post-test counselling would you consider the process as goal oriented?	Would not consider 1<br>Might or might not consider 2<br>Definitely consider 3

TPE1076: What do you think about counselling and testing for STI/HIV at university? Please, feel free to express your personal thoughts!

Appendix 4e**:**

Group interview guide

Name of the moderator ________________________________ Interview date: __/___/202__

Collection setting (university/faculty) ________/______________________________________

Group Identification Number ______________

Section 1: Introduction

Good morning and welcome to our meeting today. Thanks for coming and join us to talk about STI/HIV prevention program at the university. My name is….(the moderator will say her/his name), and assisting me are …. And ….. (names of the assistants), we are all data collector at the UCM. We are here on behalf of the department of sexual and reproductive health, gender and HIV-UCM, to get some information from first year students about your experiences, motivations and barriers that students may face to follow recommended sexual health behaviors. The department would like to know experiences lived, motivations toward making decision and barriers to engage recommended sexual behavior. We are having discussion with different groups of students around the UCM faculties.

You were invited to take part of this meeting because you have participated in the cohort and have completed yours follow up visits, so we believe you are familiar with all activities running within the program, and you are students at UCM.

We would like to let you know that there are no wrong answers, but instead, differing points of views or feelings. Please feel free to share your personal point of view, even if it is different from what others feel about. Remember we are interested in both positive and negative aspects, off cause the negative issues are the most important.

We also would like to let you know that we are recording the conversation, because we do not want to miss any of your words. You can often say very helpful words during the conversation, but we can't write fast enough to get them over, so one person speaking at time. We will participate on a first name basis during the session, but we will not use any names in our reports. Feel assured that your confidentiality is our obligation. The results will be used in future to help with strengthen the program.

Please make sure that your phone is turned off or in silence mode. If can't and you have to respond to a call, please do so as quietly as possible and come back as quickly as you can. My role as moderator is to guide de conversation! You are free to talk to each other!

Well, let's start with the session, but before, we have to place our name cards in front, so that we can refer to each other easily. Then let's tell each other around the table our name and what is your course.

Section 2: Basic questions

IGQ101: What did you feel about being exposed to STI/HIV prevention at the university?

IGQ102: Can you tell me what did you like/dislike from the program?

IGQ103: What do you think made the program interesting/or not interesting?

IGQ104: Can you tell me your experience about decision to use condom?

IGQ106: Tell me what the most important reasons are for you to decide using condom consistently.

IGQ107: What did you feel or think about negotiation to use condom?

IGQ108: Can you tell me your experience regarding following a calendar for STI/HIV screening?

IGQ9: Tell me what you think are the most important reasons for you doing regular STI/HIV screening.

IGQ110: If you have the opportunity to talk to the manager of the SRH, gender and HIV department, what would you suggest to improve the program?

Section 3: Brief summary of the session

IGQ111: Is this summary clear for everyone?

IGQ112: Have we missed anything?
